# Supplementary material for: Lengthening adalimumab dosing interval in quiescent Crohn’s disease patients: protocol for the pragmatic randomised non-inferiority LADI study
Source: BMJ Open. 2020 May 26;10(5):e035326. doi: 10.1136/bmjopen-2019-035326 (PMC7259868; doi:10.1136/bmjopen-2019-035326)
Supplement: Supplementary data [file bmjopen-2019-035326supp002.pdf]

Supplementary file 2: Subject Consent Form

Step-by-step extension of the adalimumab interval in patients with Crohn's disease

- I have read the subject information form. I was also able to ask questions. My questions have been answered to my satisfaction. I had enough time to decide whether to participate.
- I know that participation is voluntary. I know that I may decide at any time not to participate after all or to withdraw from the study. I do not need to give a reason for this.
- I give permission for my GP to be informed that I am participating in this study
- I know that some people may have access to all my data to verify the study. These people are listed in this information sheet. I consent to the inspection by them.
- I give permission for sending my HealthBeacon data to the study team.
- I agree that my GP and treating specialist will be informed of coincidental findings that (may) be of interest for my health.
- I give permission for the collection and use of my data and body material to answer the research question in this study.
- I give permission for keeping my data at the research location for 25 years.
- I give permission for registration of observational data during 2 years after the study period.
- I **do** / **do not*** consent to keeping my bodily material 15 years after this study and to use this later for other research, as indicated in the information sheet.
- I **do** / **do not*** consent to being contacted again after this study for a follow-up study.
- I want to participate in this study.

Name of study subject:
Signature: Date: __ / __ / __

I hereby declare that I have fully informed this study subject about this study.

If information comes to light during the course of the study that could affect the study subject's consent, I will inform him/her of this in a timely fashion.

Name of investigator (or his/her representative):
Signature: Date:__ / __ / __

Additional information was given by:
Name:
Job title:
Signature: Date:__ / __ / __

----

* Delete as appropriate.

Smits LJT, *et al. BMJ Open* 2020; 10:e035326. doi: 10.1136/bmjopen-2019-035326

The study subject will receive the full information sheet, together with a signed copy of the consent form.

Smits LJT, *et al. BMJ Open* 2020; 10:e035326. doi: 10.1136/bmjopen-2019-035326
