# Supplementary material for: Lengthening adalimumab dosing interval in quiescent Crohn’s disease patients: protocol for the pragmatic randomised non-inferiority LADI study
Source: BMJ Open. 2020 May 26;10(5):e035326. doi: 10.1136/bmjopen-2019-035326 (PMC7259868; doi:10.1136/bmjopen-2019-035326)
Supplement: Author's manuscript [file bmjopen-2019-035326.draft_revisions.pdf]

BMJ Open

BMJ Open is committed to open peer review. As part of this commitment we make the peer review history of every article we publish publicly available.

When an article is published we post the peer reviewers' comments and the authors' responses online. We also post the versions of the paper that were used during peer review. These are the versions that the peer review comments apply to.

The versions of the paper that follow are the versions that were submitted during the peer review process. They are not the versions of record or the final published versions. They should not be cited or distributed as the published version of this manuscript.

BMJ Open is an open access journal and the full, final, typeset and author-corrected version of record of the manuscript is available on our site with no access controls, subscription charges or pay-per-view fees (http://bmjopen.bmj.com).

If you have any questions on BMJ Open's open peer review process please email info.bmjopen@bmj.com

BMJ Open

Lengthening Adalimumab Dosing Interval in quiescent Crohn's disease patients: design of the LADI study, a pragmatic randomised non-inferiority trial.

Journal:	*BMJ Open*
Manuscript ID	bmjopen-2019-035326
Article Type:	Protocol
Date Submitted by the Author:	28-Oct-2019
Complete List of Authors:	Smits, Lisa; Radboudumc, Department of Gastroenterology and Hepatology<br>Pauwels, Renske Wilhelmina Maria; Erasmus Medical Center, Department of Gastroenterology and Hepatology<br>Kievit, Wietske; Radboudumc, Department for Health Evidence<br>Jong, D; Radboudumc, Department of Gastroenterology and Hepatology<br>De Vries, Annemarie; Erasmus Medical Center, Department of Gastroenterology and Hepatology<br>hoentjen, F; Radboudumc, Department of Gastroenterology and Hepatology<br>van der Woude, Janneke; Erasmus Medical Center, Department of Gastroenterology and Hepatology
Keywords:	Inflammatory bowel disease < GASTROENTEROLOGY, Gastroenterology < INTERNAL MEDICINE, Adverse events < THERAPEUTICS, Clinical trials < THERAPEUTICS, HEALTH ECONOMICS, STATISTICS & RESEARCH METHODS

SCHOLARONE™
Manuscripts

I, the Submitting Author has the right to grant and does grant on behalf of all authors of the Work (as defined in the below author licence), an exclusive licence and/or a non-exclusive licence for contributions from authors who are: i) UK Crown employees; ii) where BMJ has agreed a CC-BY licence shall apply, and/or iii) in accordance with the terms applicable for US Federal Government officers or employees acting as part of their official duties; on a worldwide, perpetual, irrevocable, royalty-free basis to BMJ Publishing Group Ltd ("BMJ") its licensees and where the relevant Journal is co-owned by BMJ to the co-owners of the Journal, to publish the Work in this journal and any other BMJ products and to exploit all rights, as set out in our licence.

The Submitting Author accepts and understands that any supply made under these terms is made by BMJ to the Submitting Author unless you are acting as an employee on behalf of your employer or a postgraduate student of an affiliated institution which is paying any applicable article publishing charge ("APC") for Open Access articles. Where the Submitting Author wishes to make the Work available on an Open Access basis (and intends to pay the relevant APC), the terms of reuse of such Open Access shall be governed by a Creative Commons licence – details of these licences and which Creative Commons licence will apply to this Work are set out in our licence referred to above.

Other than as permitted in any relevant BMJ Author's Self Archiving Policies, I confirm this Work has not been accepted for publication elsewhere, is not being considered for publication elsewhere and does not duplicate material already published. I confirm all authors consent to publication of this Work and authorise the granting of this licence.

For peer review only

Lengthening Adalimumab Dosing Interval in quiescent Crohn's disease patients: design of the LADI study, a pragmatic randomised non-inferiority trial.

L.J.T. Smits[1*], R.W.M. Pauwels[2*], W. Kievit[3], D.J. de Jong[1], A.C. de Vries[2], F. Hoentjen[1#], C.J. van der Woude[2#]

* Authors share first authorship
\# Authors share last authorship
1. Radboud UMC, Department of Gastroenterology and Hepatology, Nijmegen, The Netherlands
2. Erasmus MC, Department of Gastroenterology and Hepatology, Rotterdam, The Netherlands
3. Radboud UMC, Department for Health Evidence, Nijmegen, The Netherlands

Short title: Adalimumab tapering in Crohn's disease

Correspondence
C.J. van der Woude
e-mail: c.vanderwoude@erasmusmc.nl
telephone: 0031 10 703 3020
Erasmus MC, Department of Gastroenterology and Hepatology
P.O. Box 2040
3000 CA Rotterdam
The Netherlands

Word count

4503

For peer review only

ABSTRACT

Introduction: Adalimumab is effective for maintenance of remission in patients with Crohn's disease (CD) at a dose of 40mg subcutaneously every 2 weeks. However, adalimumab is associated with (long-term) adverse events and is costly. Cohort studies showed that interval lengthening may be a successful treatment strategy in a significant proportion of CD patients. The aim of this study is to demonstrate non-inferiority and cost-effectiveness of disease activity guided adalimumab injection interval lengthening compared to standard dosing of every other week (EOW).

Methods and analysis: The LADI study is a pragmatic, multicentre, open label, randomised controlled non-inferiority trial. 174 CD patients on adalimumab maintenance therapy in long-term (> 9 months) clinical and biochemical remission will be included (C-reactive protein (CRP) ≤10 mg/ml, fecal calprotectin (FC) ≤150 mg/kg, Harvey-Bradshaw Index (HBI) <5). Patients will be randomised 2:1 into the intervention (adalimumab interval lengthening) or control group (adalimumab EOW). The intervention group will lengthen the adalimumab administration interval to every 3 weeks, and after 24 weeks to every 4 weeks. Clinical and biochemical disease activity is monitored every 12 weeks by physician global assessment, HBI, CRP and FC. In case of disease flare, dosing will be increased.

Primary outcome: Non-inferiority in cumulative incidence of persistent (>8 weeks) disease flares in 48 weeks of follow-up. A flare is defined as two of three of the following criteria; FC >250 μg/g, CRP≥10 mg/L, HBI ≥5. Non-inferiority margin is 15%. Secondary outcomes include cumulative incidence of transient flares, adverse events, predictors for successful dose reduction and cost-effectiveness.

Ethics and dissemination: The study is approved by the Medical Ethics Committee Arnhem-Nijmegen, the Netherlands (registration number NL58948.091.16). Results will be published in peer-reviewed journals and presented at international conferences.
Trial registration: EudraCT: 2016-003321-42. Registered on 26 September 2016.
Clinicaltrials.gov: NCT03172377. Registered on 1 June 2017

Keywords

Crohn's disease; anti-TNF; adalimumab; calprotectin; dose reduction; interval lengthening; non-inferiority; cost-effectiveness; inflammatory bowel disease

For peer review only

STRENGTHS AND LIMITATIONS OF THIS STUDY

- The LADI study is the first randomised controlled trial that investigates adalimumab interval lengthening in Crohn's disease patients
- This pragmatic study is clinically relevant and results can easily be implemented in daily practice
- The National Crohn and colitis patients organisation is involved and patient-reported outcomes are included
- The study is not blinded

For peer review only

BACKGROUND

Crohn's disease (CD) is a chronic inflammatory disease of the gastrointestinal tract, characterized by a relapsing and remitting disease course. Patients show an abnormal mucosal immune response, resulting from an interplay of genetic susceptibility, environmental factors and the intestinal microflora.[1] Treatment consists of immunosuppressive medication, including monoclonal antibodies that block tumor necrosis factor alpha (anti-TNF); such as infliximab, adalimumab and certolizumab. Adalimumab is a humanised anti-TNF antibody that is effective as both induction and maintenance therapy for CD.[2-4] Adalimumab is administered by subcutaneous (sc) injection and an induction dose of 80 mg (week 0) and 40 mg (week 2) or 160mg (week 0) and 80mg (week 2) are generally used, followed by 40mg every 2 weeks.[5]

Although adalimumab is generally safe, side effects do occur. The risk of (opportunistic) infections is increased, especially in combination with immunosuppressive therapies, most often thiopurines or methotrexate.[6-9] A recently published review on long term safety of adalimumab (n=3606 CD patients) showed a high absolute risk of any infection of 119 events per 100 patient years (PYs) and a risk of serious infection of 6.7/100 PYs in this selected trial-population with relatively low comorbidity.[10] The incidence rate of injection site reactions (ISR: local pain and swelling) was 7.7/100 PYs).[10] In addition, several reports show an increased risk of skin cancer (both melanoma and non-melanoma skin cancer), especially in combination with thiopurines.[6 7 9 11 12] In addition to potential side effects, the costs of adalimumab are significant. Before the introduction of biosimilars, the costs of anti-TNF in the Netherlands were €15.000 – 30.000 per CD patient annually.[13 14] Anti-TNF including adalimumab is expected to continue to be the main cost driver of CD management for several reasons. First, the number of CD patients is increasing in the Netherlands.[15] Secondly, recent data stimulate an early use of anti-TNF with an accelerated step-up or top-down approach in combination with treat-to-target (mucosal healing), to prevent bowel damage.[16] Thirdly, the entry of lower cost biosimilars will possibly cause physicians to preferentially prescribe anti-TNF treatment, which will increase its use.[17-19]

Discontinuation of adalimumab therapy in CD patients in stable clinical remission is a clinical strategy that may aid in reducing the risk of side effects, costs, and avoid prolonged immunosuppression during a quiescent disease course. However, in a large meta-analysis on individual patient data (n=1264, i.e. including the landmark study by Louis et al.[20]) on cessation of anti-TNF therapy, approximately 37% of the patients had a relapse in one year, and 52% after two years of follow-up (Pauwels et al., unpublished data). Therefore, an alternative strategy of dose reduction of adalimumab rather than discontinuation may be

For peer review only

considered. In RA, the DRESS study concluded that disease activity guided dose reduction of anti-TNF is non-inferior and cost-effective, compared to maintaining regular dosing.[21 22] However, extrapolation of these results to CD is questionable, since RA patients generally use different concomitant medication, suffer from different comorbidities and anti-TNF shows different pharmacodynamic characteristics in RA patients.[23 24] In CD, adalimumab dose reduction is uncommon in daily practice. Only two retrospective cohort studies (n=46+40) reported CD patients who used adalimumab 40mg every three weeks (ETW).[25 26] After a median follow-up of 16 and 24 months, respectively 63% and 65% remained in clinical remission.

The aim of this randomised controlled trial is to demonstrate non-inferiority and cost-effectiveness of disease activity guided adalimumab injection interval lengthening compared to standard of care (continued EOW dosing) in maintaining remission in CD. In this paper we describe the study design as well as potential pitfalls and outcomes.

For peer review only

OBJECTIVE

Primary objective

- To demonstrate non-inferiority of disease activity guided adalimumab injection interval lengthening compared to adalimumab EOW dosing (standard of care) in CD patients in stable disease remission at 48 weeks of follow-up. Non-inferiority is reached if the difference in cumulative incidence of persistent flares not exceeds the non-inferiority margin. A persistent flare is defined as two of three of the following criteria, persisting for > 8 weeks despite dose escalation of adalimumab:
    - Fecal calprotectin (FC) >250 µg/g
    - C-reactive protein (CRP) ≥10 mg/L
    - Harvey-Bradshaw Index (HBI) ≥5

Secondary objectives

- To report the proportion of patients that had successful interval lengthening, defined as the absence of a disease flare, while treated with adalimumab ETW or EFW, at week 48.
- To identify factors that are associated with successful interval lengthening (e.g. baseline patient and treatment characteristics, FC, CRP, adalimumab drug levels and antibodies to adalimumab).
- To compare the cumulative incidence of patients with a transient flare (duration ≤8 weeks) between the intervention and control group at week 48.
- To compare the proportion of patients that used budesonide, prednisone or other immunomodulators in order to treat a (transient) flare.
- To compare the proportion of patients in clinical and biochemical remission between the intervention and control group at week 48. Remission is defined as a HBI <5, FC <150 µg/g and CRP <10 mg/L. In case disease activity is assessed with endoscopy or Magnetic-Resonance-Imaging (MRI) scan, that conclusion overrules our definition.
- To compare inflammatory bowel disease (IBD)-specific quality of life by the short-IBD questionnaire (SIBDQ)) between the intervention and control group every 12 weeks during follow-up.
- To compare disease activity by HBI and patient reported outcome (PRO-2) between the intervention and control group every 12 weeks during follow-up.
- To compare medical consumption (by iMTA MCQ questionnaire) and work productivity (by iMTA PCQ questionnaire) between the intervention and control group until week 48, in order to calculate the decremental cost effectiveness ratio of this interval lengthening strategy.

For peer review only

- To compare the rates of (serious) adverse events ((S)AEs) that are (possibly) related to adalimumab and the rates of (S)AEs that are (possibly) related to adalimumab interval lengthening between the intervention and control group, expressed as events/ 100 PYs of follow-up.
- To compare adalimumab use between the intervention and control group, including the cumulative dose during follow-up, the proportion of patients that uses adalimumab ETW and every four weeks (EFW).

For peer review only

METHODS

This protocol includes the standard protocol items recommended for interventional trials according to the SPIRIT guidelines (Supplementary table A).[27]

Design

This randomised controlled trial is currently being performed at the departments of Gastroenterology and Hepatology in 23 hospitals in the Netherlands, including both academic and non-academic centres. The aim of the adalimumab interval lengthening strategy is to minimize the amount of adalimumab use while maintaining remission in CD. Therefore, longer adalimumab intervals will be compared with adalimumab EOW (standard of care) in a non-inferiority design (to show the same effect is maintained with a dose reduction strategy), instead of a superiority design, which is used to demonstrate that an intervention leads to superior outcomes than the standard of care. The rationale behind a non-inferiority design is that benefits may be present in other areas (i.e. fewer side effects, lower costs) so that the intervention would be preferred if its efficacy is not worse.
The date of the first enrolment was 3 May 2017. The study is approved by the Medical Ethical Committee (METC) Arnhem-Nijmegen (registration number NL58948.091.16). Important protocol modifications are assessed and approved by the METC, and reported to participating investigators. The most recent study protocol version 3.3 (July 2018) is presented in this manuscript. The LADI study has been registered at clinicaltrials.gov (NCT03172377) and the Dutch trial register (NTRID6417). A data safety monitoring board (DSMB) is installed in order to independently assess the efficacy and safety of the study intervention and to monitor the overall conduct of the trial. Data of all participating centres will be collected by electronic case-report forms (CRF's) and monitored following good clinical practice (GCP) guidelines. The collected data will be entered in Castor, an electronic database set up for clinical trials (https://www.castoredc.com). Data will be coded and kept based on the rules for GCP by certified personnel. Prior to enrollment, all patients have to sign informed consent.

Patient and Public Involvement

The study was designed in collaboration with the Dutch Crohn's and colitis patient organisation (CCUVN) in order to optimise patient participation. We based our study design on the results of a *biological focus group* by members of the CCUVN. This focus group showed that patients do accept a reduction of the dose of their biological agent. Additionally, based on previous interactions with the CCUVN, we have included patient focused outcomes in our study, such as the quality of life and PRO-2.

For peer review only

In- and exclusion criteria

All adult CD patients with colonic and/or distal ileal and/or proximal CD, who are treated with adalimumab 40 mg every 2 weeks at a stable dose, at least 9 months in steroid-free clinical remission and not scheduled for CD-related surgery, are eligible for participation.[28] Remission is defined as a HBI <5, FC <150 μg/g and CRP <10 mg/L. The current guidelines from the European Crohn's and Colitis Organisation suggest to use CRP <10 mg/l for the definition of disease remission [5]. Endoscopic assessment prior to enrollment is not mandatory, however if an ileocolonoscopy was performed before the start of the study and demonstrated complete mucosal healing (Simple Endoscopic Score-CD <3 or no ulcerations), a FC<250 μg/g is accepted as inclusion criterium. Permitted concomitant CD therapies are: aminosalicylates, azathioprine, 6-mercatopurine, methotrexate and thioguanine at a stable dose for 12 weeks. Patients with arthralgia will be included, however inflammatory arthritis is an exclusion criterium, as this can provide elevated inflammatory markers. Furthermore, patients with active draining fistulas are excluded. Other exclusion criteria are pregnancy or lactation and other significant medical conditions that might interfere with this study (such as a current/recent malignancy, immunodeficiency syndromes and psychiatric illness), or when it is to be expected that the outcome cannot be measured (short life expectancy, planned major surgery, language issues).

Study groups

Control group

The control group continues the maintenance adalimumab sc treatment 40mg EOW. Treatment decisions are made at the discretion of the treating physician. Of note, dose reduction beyond 40 mg per two weeks is currently not recommended according to national guidelines.[29] Patients follow a standardized protocol based on the tight control/treat-to-target principle in order to maintain low disease activity.[16]

Intervention group

Adalimumab interval will be lengthened through a stepwise disease activity guided manner.

Step 1: Upon inclusion, the interval will be prolonged to ETW.

Step 2: After week 24, patients in remission will lengthen their dosing interval to EFW.

Step 3: If adalimumab interval lengthening leads to a confirmed flare, patients will return to the preceding effective interval (Figure 2). If a flare is not objectively confirmed, patients are advised to continue adalimumab in their study-interval. However, interval reduction is accepted if patients really want this as this situation reflects daily clinical practice.

For peer review only

In contrast to the DRESS study, the discontinuation of therapy after successful de-escalation to 40 mg EFW is not implemented in the study protocol.[21] Total follow-up time will be 48 weeks. Follow-up visits and outcome measurements are similar to the control group.

Co-intervention

The use of previously mentioned concomitant medication is allowed and must be documented on the CRF (stating type, dosage and duration). If possible, existing concomitant medication should not be changed during the study.

If patients experience worsening of symptoms in between visits, they must contact the outpatient clinic. For further treatment of the flare, patients in the control arm are referred to their treating physician. In the intervention arm, patients will return to the preceding effective adalimumab dosing interval (Figure 2). The decision to start concomitant therapy remains at the discretion of the treating physician.

Secondary outcome measurements

- Quality of life

For assessment of quality of life, we will use the short IBDQ, which is a validated and disease-specific questionnaire.[30]

- Patient reported disease activity

We will use the only validated IBD patient-reported outcome measure, ‘PRO-2’, consisting of reported diarrhea and abdominal pain.[31]

- Factors associated with successful dose reduction

Factors which are possibly related to successful dose reduction include: baseline patient and treatment characteristics, adalimumab drug levels (µg/mL) and antibodies (AU/mL), clinical (physician global assessment (PGA), HBI) and laboratory results (FC (µg/g), CRP (mg/L), haemoglobin (mmol/L), leucocytes (10^9/L), platelets (10^9/L), albumin (g/L)).

- Safety

AEs and SAEs are registered during follow-up. All SAEs are reported to the METC Arnhem-Nijmegen.

- Cost-effectiveness

The impact of dose reduction on the quality of life of patients will be assessed by the EQ-5D at 24 and 48 weeks following randomisation, compared to baseline. The EQ-5D utility will be

For peer review only

used to derive a quality-adjusted life year (QALY) estimate for each patient according to the trapezium rule.[32 33]

Assessments

Enrolled patients will visit the outpatient clinic every 24 weeks. If preferred by the patient or treating physician, the evaluations at week 12 and 36 can take place as outpatient clinic visit as well. Every 12 weeks, laboratory tests (e.g. FC, CRP, haemoglobin and albumin) will be performed. At week 0, 24 and 48 serum samples are stored for measurement of adalimumab drug levels and antibodies to adalimumab. Additionally, patients in both arms will be interviewed via telephone every 6 weeks in between clinical visits to assess for adverse events, symptoms and potential disease activity. If such an interview suggests a disease flare, patients must visit the outpatient clinic in order to undergo complete disease activity assessment and laboratory and FC tests. If patients have a flare at week 48, disease activity will be monitored until disease remission, in order to define the flare as persistent- or transient flare. In addition, study questionnaires are automatically sent via Castor every 6 weeks. During follow-up, patients note the dates of their adalimumab injections in a diary. An overview of all visits and assessments is depicted in Table 1, Figure 1 and Figure 2.

For peer review only

Table 1. SPIRIT schedule of enrolment, interventions, and assessments

	STUDY PERIOD											
	**Enrolment**	**Allocation**	**Follow-up**									**Extra**
**TIMEPOINT**	**$-t_1$**	**0**	w_0	w_6	w_{12}	w_{18}	w_{24}	w_{30}	w_{36}	w_{42}	w_{48}	w_e
**ENROLMENT:**												
**Eligibility screen**	X											
**Informed consent**	X											
**Allocation**		X										
**INTERVENTIONS:**												
Intervention: Lengthening adalimumab dosing interval			←	—	—	—	—	—	—	—	→	
Control: Adalimumab every other week			←	—	—	—	—	—	—	—	→	
**ASSESSMENTS:**												
Medical history	X	X										
Laboratory tests*			X		X		X		X		X	X
Fecal calprotectin			X		X		X		X		X	X
Storage of serum samples			X				X				X	X
Concomitant medication			X	X	X	X	X	X	X	X	X	X
(Serious) adverse events			X	X	X	X	X	X	X	X	X	X
Physician global assessment			X	X	X	X	X	X	X	X	X	X
HBI and PRO-2			X		X		X		X		X	X
IBD-Q and EQ5D			X		X		X		X		X	X
iMTA MCQ, -PCQ			X		X		X		X		X	X

**Hemoglobin, leukocytes, thrombocytes, albumin, C-reactive protein.*

For peer review only

HBI = Harvey Bradshaw Index, PRO-2 = Patient Reported Outcome-2, IBD-Q = Inflammatory Bowel Disease Questionnaire, EQ5D = EuroQuol 5D, iMTA MCQ = institute for Medical Technology Assessment Medical Consumption Questionnaire, PCQ = Productivity Cost Questionnaire

Randomisation, allocation concealment, stratification

Patients are randomised by the research physician using a computer-generated randomisation system (Castor). Castor uses a validated variable block randomisation model with block sizes of 6, 9 and 12. Patients will be randomised in a 2:1 ratio for the intervention or the control group, respectively. We chose 2:1 randomisation to stimulate patient inclusion, as patients have a higher chance to randomise for the intervention group. Furthermore, more determinants can be included in a prediction model for successful dose reduction.

Patients will be stratified on co-medication use (yes/no), as the incidence of flares could possibly be different with or without co-medication use. Co-medication includes azathioprine, 6-mercaptopurine, 6-thioguanine, methotrexate. Both patients and physicians are un blinded, as we aim to represent daily practice during this pragmatic study .

Sample size

The null hypothesis in non-inferiority studies is that the intervention is inferior compared to the control arm by more than the non-inferiority margin. The alternative hypothesis is that the intervention is not worse than the control by more than the non-inferiority margin. Therefore, if the null hypothesis is rejected, the alternative hypothesis that the intervention is non-inferior is accepted.[34] Based on an extrapolation of data from the DRESS study and results from a real-life CD cohort in Leuven, an estimated 15% of patients will experience the primary outcome (persistent flare) in the control arm. In the Leuven cohort, 41/156 (26%) patients discontinued adalimumab due to loss of response, despite adalimumab dose escalation.[21 35] The latter 26% was adjusted to an expected 15% for our cohort because the follow-up time in our cohort concerns 12 rather than 20 months, and our cohort is a preselected cohort of patients in long and stable remission rather than a cross-sectional cohort. In non-inferiority analyses, one-sided testing is used. Applying one sided testing, an alpha of 0.05 ($Z\alpha = 1.64$), power 1-beta 0.8 ($Z\beta = 0.84$), an non-inferiority margin of 15% and randomisation ratio of 2:1 intervention versus control resulted in n = 105 and n = 53 for intervention and control arm, respectively. Accounting for a 10% drop-out, 174 patients have to be included in total.

A non-inferiority margin of 15% means a maximum difference in persistent flare of 15% between the usual care and intervention group. We believe this strikes an acceptable balance between the potential harms of flare, and the benefits of dose reduction (fewer injections, potential for reduced risk of side effects and cost-savings). The large Nor-Switch

For peer review only

trial also used a non-inferiority margin of 15% for disease worsening during follow-up.[36] Based on this example, discussions in our study-group and approval of the protocol by the Dutch Organisation for Health Research and Development, we believe this margin is appropriate. The DRESS study used a non-inferiority margin of 20%. Although side effects/SAEs of adalimumab seem comparable in RA versus IBD, rheumatologists probably accept a higher proportion of flares because there are more alternative biological therapies available, thus a loss of effect of one biological therapy might be given less weight in RA.[37]

Planned data analysis

The primary outcome; cumulative incidence of persistent flares will be expressed as proportions in both groups. A confidence interval for the difference between study groups will be determined (adjusted for co-medication use at baseline using the Cochran-Mantel-Haenszel procedure, as this variable is used for stratification in the randomisation process[38]). The upper limit of the confidence interval will be compared with the non-inferiority margin. We will use both intention to treat and per protocol analyses, as the latter is considered the most conservative analysis for non-inferiority trials.[39] Patients in the interval lengthening group are included in the per protocol analyses if they: lengthened the adalimumab interval at least to three weeks, regardless whether they returned to a preceding effective interval in case of a disease flare. Patients in the control group are included in the per protocol analyses if they: used adalimumab EOW without consistent interval lengthening, incidental postponement of an injection during infection or around holidays is allowed. Descriptive patient (and treatment) baseline variables will be summarized as means ± SD, medians with interquartile ranges or percentages, depending on the type of measurement. Gender, BMI, age, prior medication for CD, disease duration, Montreal classification, IBD-related surgical history, comorbidity, inflammatory parameters including HBI, FC, CRP, adalimumab drug levels and antibodies to adalimumab will be reported.

The secondary continuous outcomes HBI, SIBDQ, PRO-2, adalimumab drug levels and antibody levels at 48 weeks will be analysed by either student's t-test or Mann Whitney U test depending on the type of distribution of the data. In addition, the course over time for several continuous outcomes measured at multiple time points (every 12 weeks) will be analysed using repeated measures analyses in which the outcome can be corrected for the baseline value of the specific outcome and potential confounding factors. The number of (S)AEs that are (possibly) related to adalimumab or to adalimumab interval lengthening will be reported as rates, defined as events/100 PYs of follow-up; details of these (S)AEs will be provided. In the intervention group, patient characteristics and clinical features will be analysed to predict a persistent flare. A prediction model will be developed and fitted using a univariable

For peer review only - http://bmjopen.bmj.com/site/about/guidelines.xhtml

selection based on a p-value <0.2 and a multivariable approach with backward selection. Predictive accuracy will be determined by the area under the receiver operating curve. A two-sided P-value of <0.05 is considered statistically significant. All statistical analyses will be performed by using IBM SPS Statistics 25.0.

Data analysis: Cost effectiveness

The cost analysis consists of two main parts. First, at patient level, volumes of care related to the CD care and anti-TNF therapy will be measured by means of the iMTA Medical Consumption Questionnaire (MCS). This questionnaire measures all relevant health care related costs like outpatient visits at any medical specialist, hospitalizations and imaging procedures. Loss of productivity due to illness or recovery in patients below the age of 65 will be estimated based on patient reported absences from paid (or unpaid) labor measured with the Productivity Cost Questionnaire (PCS). The second part of the cost analysis consists of determining the cost prices for each volume of consumption. The standard cost prices from the 'Dutch Guidelines for Cost Analyses' and www.medicijnkosten.nl will be used. For units of care where no standard prices are available real costs prices will be determined on the basis full cost pricing. Productivity losses will be valued by means of the friction cost method. In the end volumes of care will be multiplied with the cost prices for each volume of care to calculate costs. Because we anticipate non-inferiority of the dose reduction strategy we will primarily analyse cost-savings: direct medical cost as well as total costs (medical and non-medical costs) will be compared between intervention and control group. A possible small but acceptable loss of effect can be incorporated in the analyses by determining a decremental cost-effectiveness ratio (DCER) by dividing the difference in costs by the difference in QALYs between the groups. The DCER expresses with how much money a loss of 1 QALY is compensated. If this amount is high the decision makers may be willing to accept a loss of effect. Uncertainty in the DCER will be non-parametrically determined using bootstrap techniques (1000 replications). Results from this analysis will be presented in a scatter plot and willingness to pay (or accept) curve. Furthermore the Net Monetary Benefit (NMB) per patient will be calculated for different levels of willingness to accept (WTA) in euro's per QALY, using the formula: WTA * effect (difference in QALY) - costs. This results in the net amount of money saved, when the possible loss of QALY is corrected for, using different WTA levels per QALY.

For peer review only

DISCUSSION

Dose reduction of adalimumab in CD patients with stable disease may provide similar disease control but reduction of adverse events and costs. With this pragmatic, non-inferiority study design we aim to evaluate the outcomes of this strategy. Only two small retrospective studies reported on adalimumab 40mg ETW in CD patients.[25 26] No prospective randomised data are available. Prior studies have investigated the effect of discontinuation of anti-TNF therapy in CD.[40-43] Previous clinical trials on withdrawal of anti-TNF after a period of prolonged remission in CD patients showed a relatively consistent profile of 42% relapses after anti-TNF cessation within one year of follow-up.[40-43] Louis et al. identified risk factors for disease flare after discontinuation of infliximab in CD patients who used infliximab and thiopurine combination therapy for at least one year. Risk factors for relapse included male sex, high leukocyte counts, high CRP, high FC and low levels of hemoglobin.[20] The multicentre randomised CEASE trial (ZonMw project number 848101009) will further investigate cessation of anti-TNF. As cessation of anti-TNF therapy is a different research question with different outcome measures, uncertainty remains on factors that are associated with successful adalimumab interval lengthening and the LADI study will provide useful information for daily clinical practice.

We decided to assess non-inferiority with regard to persistent flares (persisting >8 weeks independent of treatment changes such as adalimumab dose escalation) since these are the most relevant clinical outcomes in this setting. Temporary flares (persisting <8 weeks) that resolve after appropriate treatment are less difficult to manage and are likely to occur as an acceptable result of searching for the optimal individualized treatment interval. Temporary flares will still function as relevant secondary outcome in our trial. For the definition of a flare, 2 consecutive measurements demonstrating two out of three of the following criteria; FC >250 μg/g, CRP ≥10 mg/L, HBI ≥5 are required. As it has been shown that flares are frequently temporary and occur and sometimes disappear without regimen change, a flare is only considered a flare if it is confirmed two times. For this composite endpoint we preferred to incorporate the HBI instead of the Crohn's Disease Activity Index on account of accessible clinical implementation in daily practice. In addition, FC and CRP are non-invasive, cheap and widely available biomarkers of disease activity.[44] Furthermore, FC correlates to endoscopic disease activity.[45 46] Recently, it was shown that an increase in FC can precede on the onset of clinical symptoms.[47] Indeed, due to our definition of a flare, patients without clinical symptoms can also fulfill the definition of a (biochemical) flare. In addition, the requirement of an elevation in inflammatory markers at two time points allows for the exclusion of confounders such as infections as Clostridium difficile and use of NSAIDs).

For peer review only - http://bmjopen.bmj.com/site/about/guidelines.xhtml

We decided not to include endoscopy outcomes in the inclusion criteria or primary endpoint. An endoscopic procedure is a burden for patients due to the invasive procedure and the intensive preparation. In addition, we aimed for study results that may be easily implemented in daily practice. Instead of an endoscopy, we used a combination of surrogate markers of inflammation including HBI, CRP and FC to determine clinical remission. A protocolized treatment is advised when a flare occurs (Figure 2). However, treatment choices are not mandatory and bridging therapy (including steroids) is left to the discretion of the treating physician.

For the study design, a blinded design was considered, but the development, costs and administration of placebo injections would create a formidable barrier for the study. Furthermore, an un-blinded (pragmatic) design fits best with the current ideas about the external validity of cost-effectiveness studies. This design mirrors the real-life setting which is also not blinded, with respect to costs and effects. In general, an unblinded study design could result in information and attribution bias, e.g. flares in patients in whom the dose is reduced would possible be reported sooner. Because this will not lead to an underestimation of the drawbacks of a dose reduction strategy, this bias was accepted.

Our trial will provide important insights in addition to the risk of recurrence as well as the risk of persistent flares. For example, we will collect valuable series of drug measurements of adalimumab. Although the DRESS study did not show predictive value of drug levels for the success of dose reduction, daily IBD practice does apply measurement of drug levels. It is possible that drug levels at baseline, either low or high, may predict successful dose reduction.

From a societal perspective, it is important to improve the cost-effectiveness of IBD healthcare. Patients with chronic inflammatory diseases use expensive medication for many years and there is a growing amount of new (expensive) drugs that will soon be implemented in daily clinical care. In RA and psoriasis, dose reduction trials in adalimumab treated patients are performed and in RA the feasibility of this strategy was already demonstrated and results from a Dutch nation-wide psoriasis trial will follow soon.[21 48] The recent introduction of biosimilars of adalimumab will further aid in cost reduction but the new costs of this therapy will still remain significant. Therefore, cost savings due to dose reduction will remain relevant.

In conclusion, we designed a pragmatic randomized controlled trial to assess the non-inferiority of a strategy of adalimumab dose reduction in CD patients. Accurate prediction of

For peer review only - http://bmjopen.bmj.com/site/about/guidelines.xhtml

successful tapering may aid in reduction of costs and adverse events to further improve care for CD patients.

For peer review only

Abbreviations

CD	Crohn's disease
HBI	Harvey-Bradshaw Index
CRP	c-reactive protein
FC	fecal calprotectin
ADA	antidrug antibodies
RA	rheumatoid arthritis
EOW	every other week
ETW	every three weeks
EFW	every four weeks
(S)AE	(serious) adverse event
PYs	patient years
IBD	inflammatory bowel disease
SIBDQ	short-IBD questionnaire
PRO-2	patient reported outcome-2
MCQ	medical consumption questionnaire
PCQ	productivity cost questionnaire
PGA	physician global assessment
TNF	tumor necrosis factor
METC	Medical Ethical Committee
DSMB	data safety monitoring board
CRF	case-report form
GCP	good clinical practice
QALY	quality-adjusted life year
DCER	decremental cost-effectiveness ratio
NMB	Net Monetary Benefit
WTA	willingness to accept

Acknowledgements

This study is supported by the Dutch Initiative on Crohn and Colitis (ICC), a nationwide network of IBD centres that aims at initiating and facilitating IBD research. We thank dr. A.A. den Broeder, rheumatologist at St. Maartenskliniek Nijmegen, for his invaluable help in designing the study.

Funding

The investigator initiated LADI study is supported by the Netherlands Organisation for Health Research and Development (ZonMw, Health Care Efficiency program, grant number

For peer review only

848015002). ZonMw is part of the Netherlands Organisation for Scientific Research (NWO).

Roles of protocol contributors

FH and JvdW are principal investigators and designed the LADI study. WK provided statistical expertise in clinical trial design. AdV, DdJ, RP, LS critically reviewed the study design. Study coordinators LS and RP ensure daily study management. LS and RP drafted the manuscript and all authors read, revised and approved the final manuscript.

Disclosures

L.J.T. Smits has nothing to disclose.

R.W.M. Pauwels has nothing to disclose.

W. Kievit has nothing to disclose.

D.J. de Jong received consulting fees from Synthon Pharma, Abbvie, and MSD, and travel fees from Falk Pharma, Takeda, Abbvie, MSD, Ferring, Vifor Pharma, and Cablon Medical.

A.C. de Vries has participated in advisory board and/or received financial compensation from the following companies: Jansen, Takeda, Abbvie and Tramedico.

F. Hoentjen has served on advisory boards or as speaker for Abbvie, Janssen-Cilag, MSD, Takeda, Celltrion, Teva, Sandoz and Dr Falk, and received unrestricted funding from Dr Falk, Janssen-Cilag, Abbvie and Celgene.

C.J. van der Woude received grant support from Falk Benelux and Pfizer; received speaker fees from AbbVie, Takeda, Ferring, Dr. Falk Pharma, Hospira, Pfizer; and served as a consultant for AbbVie, MSD, Takeda, Celgene, Mundipharma and Janssen.

Coordinating centres

Radboud University Medical Centre, Department of Gastroenterology and Hepatology, Nijmegen, The Netherlands

Erasmus Medical Centre, Department of Gastroenterology and Hepatology, Rotterdam, The Netherlands

Sponsor

Radboud University Medical Centre

For peer review only

P.O. Box 9101

6500 HB Nijmegen, The Netherlands

Principal investigators

F. Hoentjen, MD PhD & C.J. van der Woude, MD PhD

Participating centres and lead investigators

1. Albert Schweitzer Hospital, Dordrecht, The Netherlands; F.H.J. Wolfhagen, MD PhD, Department of Gastroenterology
2. Amphia Hospital, Breda, The Netherlands; A.G.L. Bodelier, MD PhD, Department of Gastroenterology
3. Amsterdam University Medical Centre-location AMC, Amsterdam, The Netherlands; M. Löwenberg, MD PhD, Department of Gastroenterology
4. Amsterdam University Medical Centre-location VUmc, Amsterdam, The Netherlands; N. de Boer, MD PhD, Department of Gastroenterology
5. Bernhoven Hospital, Uden, The Netherlands; I.A.M. Gisbertz, MD PhD, Department of Gastroenterology
6. Canisius-Wilhelmina Hospital, Nijmegen, The Netherlands; A.C.I.T.L. Tan, MD PhD, Department of Gastroenterology
7. Erasmus Medical Centre, Rotterdam, The Netherlands; C.J. van der Woude, MD PhD, Department of Gastroenterology
8. Elisabeth-Tweesteden Hospital, Tilburg, The Netherlands; M.W.M.D. Lutgens, MD PhD, Department of Gastroenterology
9. Flevoziekenhuis Hospital, Almere, The Netherlands; R.C. Mallant-Hent, MD PhD, Department of Gastroenterology
10. Franciscus Gasthuis & Vlietland, Rotterdam, The Netherlands; R.L. West, MD PhD, Department of Gastroenterology
11. IJsselland Hospital, Capelle aan den IJssel, The Netherlands; I. Schot, MD, & K.Robbers, MD, Department of Gastroenterology
12. Ikazia Hospital, Rotterdam, The Netherlands; P.C.J. ter Borg, MD PhD, Department of Gastroenterology
13. Jeroen Bosch Hospital, Den Bosch, The Netherlands; T. Römkens, MD PhD, Department of Gastroenterology
14. Leiden University Medical Centre, Leiden, The Netherlands; A.E. van der Meulen, MD PhD, Department of Gastroenterology
15. Maastricht University Medical Centre+, Maastricht, The Netherlands; M. Pierik, MD Phd, Department of Gastroenterology

For peer review only

16. Maxima Medical Centre, Veldhoven/Eindhoven, The Netherlands; P.J. Boekema, MD PhD, Department of Gastroenterology
17. Medisch Spectrum Twente, Enschede, The Netherlands; M.G.V.M. Russel, MD PhD, Department of Gastroenterology
18. Onze Lieve Vrouwe Gasthuis, Amsterdam, The Netherlands; J. Jansen, MD PhD, Department of Gastroenterology
19. Radboud University Medical Centre, Nijmegen, The Netherlands; F. Hoentjen, MD PhD, Department of Gastroenterology
20. Reinier de Graaf Hospital, Delft, The Netherlands; S.V. Jansen, MD, Department of Gastroenterology
21. Spaarne Gasthuis, Haarlem, The Netherlands; J.P. Kuijvenhoven, MD PhD, Department of Gastroenterology
22. University Medical Centre Utrecht, Utrecht, The Netherlands; B .Oldenburg, MD PhD, Department of Gastroenterology
23. Zuyderland Hospital, Heerlen/Sittard, The Netherlands; M.J.L. Romberg-Camps, MD PhD, Department of Gastroenterology

Data safety monitoring board

R.J.M. Brüggemann, PharmD PhD, Department of Pharmacy, Radboud University Medical Centre, Nijmegen, The Netherlands (chair)

J. in t Hout, PhD, Department for Health Evidence, Radboud University Medical Centre, Nijmegen, The Netherlands

R.J. de Knegt, MD PhD, Department of Gastroenterology and Hepatology, Erasmus Medical Centre, Rotterdam, The Netherlands

Independent expert

E.T.T.L. Tjwa, MD PhD, Department of Gastroenterology and Hepatology, Radboud University Medical Centre, Nijmegen, The Netherlands

Availability of data and materials

The dataset generated during the LADI study is available on reasonable request.

For peer review only

FIGURE LEGENDS

Figure 1. Schematic presentation of the trial design.

ADA = adalimumab, W0 = week 0, W6 = week 6, etc. Lab tests include hemoglobin, leukocytes, thrombocytes, albumin, C-reactive protein, calprotectin

Figure 2. Protocolised treatment recommendation in case of disease flare

T0 = start of possible disease flare, which can occur at any time during follow-up, T2 = 2 weeks after T0, T6-8 = 6-8 weeks after T0. Lab tests include hemoglobin, leukocytes, thrombocytes, albumin, C-reactive protein, calprotectin

For peer review only

REFERENCES

1. Torres J, Mehandru S, Colombel JF, et al. Crohn's disease. *Lancet* 2017;389(10080):1741-55. doi: 10.1016/s0140-6736(16)31711-1 [published Online First: 2016/12/05]
2. Colombel JF, Sandborn WJ, Rutgeerts P, et al. Adalimumab for maintenance of clinical response and remission in patients with Crohn's disease: the CHARM trial. *Gastroenterology* 2007;132(1):52-65. doi: 10.1053/j.gastro.2006.11.041
3. Sandborn WJ, Hanauer SB, Rutgeerts P, et al. Adalimumab for maintenance treatment of Crohn's disease: results of the CLASSIC II trial. *Gut* 2007;56(9):1232-9. doi: 10.1136/gut.2006.106781
4. Hanauer SB, Sandborn WJ, Rutgeerts P, et al. Human anti-tumor necrosis factor monoclonal antibody (adalimumab) in Crohn's disease: the CLASSIC-I trial. *Gastroenterology* 2006;130(2):323-33; quiz 591. doi: 10.1053/j.gastro.2005.11.030 [published Online First: 2006/02/14]
5. Gomollon F, Dignass A, Annese V, et al. 3rd European Evidence-based Consensus on the Diagnosis and Management of Crohn's Disease 2016: Part 1: Diagnosis and Medical Management. *Journal of Crohn's & colitis* 2017;11(1):3-25. doi: 10.1093/ecco-jcc/jjw168 [published Online First: 2016/09/24]
6. Burmester GR, Panaccione R, Gordon KB, et al. Adalimumab: Long-term safety in 23 458 patients from global clinical trials in rheumatoid arthritis, juvenile idiopathic arthritis, ankylosing spondylitis, psoriatic arthritis, psoriasis and Crohn's disease. *Ann Rheum Dis* 2013;72(4):517-24. doi: 10.1136/annrheumdis-2011-201244
7. Osterman MT, Sandborn WJ, Colombel JF, et al. Increased risk of malignancy with adalimumab combination therapy, compared with monotherapy, for Crohn's disease. *Gastroenterology* 2014;146(4):941-9. doi: 10.1053/j.gastro.2013.12.025 [published Online First: 2013/12/24]
8. Pariente B, Laharie D. Review article: why, when and how to de-escalate therapy in inflammatory bowel diseases. *Alimentary pharmacology & therapeutics* 2014;40(4):338-53. doi: 10.1111/apt.12838 [published Online First: 2014/06/25]
9. D'Haens G, Reinisch W, Panaccione R, et al. Lymphoma Risk and Overall Safety Profile of Adalimumab in Patients With Crohn's Disease With up to 6 Years of Follow-Up in the Pyramid Registry. *The American journal of gastroenterology* 2018;113(6):872-82. doi: 10.1038/s41395-018-0098-4 [published Online First: 2018/06/06]
10. Colombel JF, Sandborn WJ, Reinisch W, et al. Long-term safety of adalimumab in clinical trials in adult patients with Crohn's disease or ulcerative colitis. *Alimentary pharmacology & therapeutics* 2018;47(2):219-28. doi: 10.1111/apt.14420 [published Online First: 2017/11/22]
11. McKenna MR, Stobaugh DJ, Deepak P. Melanoma and non-melanoma skin cancer in inflammatory bowel disease patients following tumor necrosis factor-alpha inhibitor monotherapy and in combination with thiopurines: analysis of the Food and Drug Administration Adverse Event Reporting System. *Journal of gastrointestinal and liver diseases : JGLD* 2014;23(3):267-71. doi: 10.15403/jgld.2014.1121.233.mrmk [published Online First: 2014/10/01]
12. Long MD, Martin CF, Pipkin CA, et al. Risk of melanoma and nonmelanoma skin cancer among patients with inflammatory bowel disease. *Gastroenterology* 2012;143(2):390-99.e1. doi: 10.1053/j.gastro.2012.05.004 [published Online First: 2012/05/16]
13. van der Valk ME, Mangen MJ, Leenders M, et al. Healthcare costs of inflammatory bowel disease have shifted from hospitalisation and surgery towards anti-TNFalpha therapy: results from the COIN study. *Gut* 2014;63(1):72-9. doi: 10.1136/gutjnl-2012-303376 [published Online First: 2012/11/09]
14. van der Valk ME, Mangen MJ, Severs M, et al. Evolution of Costs of Inflammatory Bowel Disease over Two Years of Follow-Up. *PLoS One* 2016;11(4):e0142481. doi: 10.1371/journal.pone.0142481 [published Online First: 2016/04/23]
15. van den Heuvel TRA, Jeuring SFG, Zeegers MP, et al. A 20-Year Temporal Change Analysis in Incidence, Presenting Phenotype and Mortality, in the Dutch IBDSL Cohort-Can Diagnostic Factors Explain the Increase in IBD Incidence? *Journal of*

For peer review only - http://bmjopen.bmj.com/site/about/guidelines.xhtml

Crohn's & colitis 2017;11(10):1169-79. doi: 10.1093/ecco-jcc/jjx055 [published Online First: 2017/04/22]
16. Colombel JF, Panaccione R, Bossuyt P, et al. Effect of tight control management on Crohn's disease (CALM): a multicentre, randomised, controlled phase 3 trial. *Lancet* 2018;390(10114):2779-89. doi: 10.1016/s0140-6736(17)32641-7 [published Online First: 2017/11/04]
17. Brodszky V, Rencz F, Pentek M, et al. A budget impact model for biosimilar infliximab in Crohn's disease in Bulgaria, the Czech Republic, Hungary, Poland, Romania, and Slovakia. *Expert review of pharmacoeconomics & outcomes research* 2016;16(1):119-25. doi: 10.1586/14737167.2015.1067142 [published Online First: 2015/07/15]
18. Kuenzig ME, Benchimol EI, Lee L, et al. The Impact of Inflammatory Bowel Disease in Canada 2018: Direct Costs and Health Services Utilization. *Journal of the Canadian Association of Gastroenterology* 2019;2(Suppl 1):S17-s33. doi: 10.1093/jcag/gwy055 [published Online First: 2019/07/12]
19. Bots S, Hoekman DR, Benninga MA, et al. Patterns of anti-TNF use and associated treatment outcomes in inflammatory bowel disease patients: Results from an analysis of dutch health insurance claims data. *Gastroenterology* 2017;152(5):S58.
20. Louis E, Mary JY, Vernier-Massouille G, et al. Maintenance of remission among patients with Crohn's disease on antimetabolite therapy after infliximab therapy is stopped. *Gastroenterology* 2012;142(1):63-70 e5; quiz e31. doi: 10.1053/j.gastro.2011.09.034
21. van Herwaarden N, van der Maas A, Minten MJ, et al. Disease activity guided dose reduction and withdrawal of adalimumab or etanercept compared with usual care in rheumatoid arthritis: open label, randomised controlled, non-inferiority trial. *Bmj* 2015;350:h1389. doi: 10.1136/bmj.h1389
22. Kievit W, van Herwaarden N, van den Hoogen FH, et al. Disease activity-guided dose optimisation of adalimumab and etanercept is a cost-effective strategy compared with non-tapering tight control rheumatoid arthritis care: analyses of the DRESS study. *Annals of the rheumatic diseases* 2016;75(11):1939-44. doi: 10.1136/annrheumdis-2015-208317 [published Online First: 2016/01/15]
23. Papamichael K, Van Stappen T, Jairath V, et al. Review article: pharmacological aspects of anti-TNF biosimilars in inflammatory bowel diseases. *Alimentary pharmacology & therapeutics* 2015;42(10):1158-69. doi: 10.1111/apt.13402 [published Online First: 2015/09/15]
24. Tilg H, Moschen A, Kaser A. Mode of function of biological anti-TNF agents in the treatment of inflammatory bowel diseases. *Expert Opin Biol Ther* 2007;7(7):1051-9. doi: 10.1517/14712598.7.7.1051 [published Online First: 2007/08/02]
25. Pouillon L, Lamoureux A, Pineton de Chambrun G, et al. Dose de-escalation to adalimumab 40mg every three weeks in patients with inflammatory bowel disease-A multicenter, retrospective, observational study. *Digestive and liver disease : official journal of the Italian Society of Gastroenterology and the Italian Association for the Study of the Liver* 2019;51(2):236-41. doi: 10.1016/j.dld.2018.10.022 [published Online First: 2018/12/07]
26. Van Steenbergen S, Bian S, Vermeire S, et al. Dose de-escalation to adalimumab 40 mg every 3 weeks in patients with Crohn's disease - a nested case-control study. *Alimentary pharmacology & therapeutics* 2017;45(7):923-32. doi: 10.1111/apt.13964 [published Online First: 2017/02/07]
27. Chan AW, Tetzlaff JM, Gotzsche PC, et al. SPIRIT 2013 explanation and elaboration: guidance for protocols of clinical trials. *Bmj* 2013;346:e7586. doi: 10.1136/bmj.e7586 [published Online First: 2013/01/11]
28. ECCO obo, Gionchetti P, Gomollón F, et al. 3rd European Evidence-based Consensus on the Diagnosis and Management of Crohn’s Disease 2016: Part 1: Diagnosis and Medical Management. *Journal of Crohn's and Colitis* 2016;11(1):3-25. doi: 10.1093/ecco-jcc/jjw168
29. van Bodegraven AA, van Everdingen JJ, Dijkstra G, et al. [Guideline 'Diagnosis and treatment of inflammatory bowel disease in adults'. I. Diagnosis and treatment]. *Nederlands tijdschrift voor geneeskunde* 2010;154:A1899. [published Online First: 2010/10/30]

For peer review only - http://bmjopen.bmj.com/site/about/guidelines.xhtml

30. Irvine EJ, Zhou Q, Thompson AK. The Short Inflammatory Bowel Disease Questionnaire: a quality of life instrument for community physicians managing inflammatory bowel disease. CCRPT Investigators. Canadian Crohn's Relapse Prevention Trial. *The American journal of gastroenterology* 1996;91(8):1571-8. [published Online First: 1996/08/01]
31. Khanna R, Zou G, D'Haens G, et al. A retrospective analysis: the development of patient reported outcome measures for the assessment of Crohn's disease activity. *Alimentary pharmacology & therapeutics* 2015;41(1):77-86. doi: 10.1111/apt.13001 [published Online First: 2014/10/29]
32. Janssen MF, Pickard AS, Golicki D, et al. Measurement properties of the EQ-5D-5L compared to the EQ-5D-3L across eight patient groups: a multi-country study. *Quality of life research : an international journal of quality of life aspects of treatment, care and rehabilitation* 2013;22(7):1717-27. doi: 10.1007/s11136-012-0322-4
33. EuroQol G. EuroQol--a new facility for the measurement of health-related quality of life. *Health policy* 1990;16(3):199-208.
34. Flight L, Julious SA. Practical guide to sample size calculations: non-inferiority and equivalence trials. *Pharmaceutical statistics* 2016;15(1):80-9. doi: 10.1002/pst.1716 [published Online First: 2015/11/26]
35. Karmiris K, Paintaud G, Noman M, et al. Influence of trough serum levels and immunogenicity on long-term outcome of adalimumab therapy in Crohn's disease. *Gastroenterology* 2009;137(5):1628-40. doi: 10.1053/j.gastro.2009.07.062 [published Online First: 2009/08/12]
36. Jorgensen KK, Olsen IC, Goll GL, et al. Switching from originator infliximab to biosimilar CT-P13 compared with maintained treatment with originator infliximab (NOR-SWITCH): a 52-week, randomised, double-blind, non-inferiority trial. *Lancet* 2017;389(10086):2304-16. doi: 10.1016/s0140-6736(17)30068-5 [published Online First: 2017/05/16]
37. Burmester GR, Panaccione R, Gordon KB, et al. Adalimumab: long-term safety in 23 458 patients from global clinical trials in rheumatoid arthritis, juvenile idiopathic arthritis, ankylosing spondylitis, psoriatic arthritis, psoriasis and Crohn's disease. *Annals of the rheumatic diseases* 2013;72(4):517-24. doi: 10.1136/annrheumdis-2011-201244 [published Online First: 2012/05/09]
38. Kahan BC, Morris TP. Reporting and analysis of trials using stratified randomisation in leading medical journals: review and reanalysis. *Bmj* 2012;345:e5840. doi: 10.1136/bmj.e5840 [published Online First: 2012/09/18]
39. E9 I. Statistical principles for clinical trials. Note for guidance on statistical principles for clinical trials. *EMA*;CPMP/ICH/363/96
40. Gisbert JP, Marín AC, Chaparro M. The risk of relapse after Anti-TNF discontinuation in inflammatory bowel disease: Systematic review and meta-analysis. *Am J Gastroenterol* 2016;111(5):632-47. doi: 10.1038/ajg.2016.54
41. Kennedy NA, Warner B, Johnston E, et al. Anti-TNF withdrawal in IBD: Relapse and recapture rates and predictive factors from 160 patients in a pan-UK study. *J Crohn's Colitis* 2015;9:S41-S42. doi: 10.1093/ecco-jcc/jju027
42. Louis E, Mary JY, Verniermassouille G, et al. Maintenance of remission among patients with Crohn's disease on antimetabolite therapy after infliximab therapy is stopped. *Gastroenterology* 2012;142(1):63-70.e5. doi: 10.1053/j.gastro.2011.09.034
43. Casanova MJ, Chaparro M, García-Sánchez V, et al. Evolution after Anti-TNF Discontinuation in Patients with Inflammatory Bowel Disease: A Multicenter Long-Term Follow-Up Study. *Am J Gastroenterol* 2017;112(1):120-31. doi: 10.1038/ajg.2016.569
44. Ma C, Battat R, Parker CE, et al. Update on C-reactive protein and fecal calprotectin: are they accurate measures of disease activity in Crohn's disease? *Expert Rev Gastroenterol Hepatol* 2019:1-12. doi: 10.1080/17474124.2019.1563481 [published Online First: 2019/02/23]
45. D'Haens G, Ferrante M, Vermeire S, et al. Fecal calprotectin is a surrogate marker for endoscopic lesions in inflammatory bowel disease. *Inflammatory bowel diseases* 2012;18(12):2218-24. doi: 10.1002/ibd.22917

For peer review only - http://bmjopen.bmj.com/site/about/guidelines.xhtml

46. Kennedy NA, Jones GR, Plevris N, et al. Association Between Level of Fecal Calprotectin and Progression of Crohn's Disease. *Clin Gastroenterol Hepatol* 2019 doi: 10.1016/j.cgh.2019.02.017 [published Online First: 2019/02/18]
47. Ferreiro-Iglesias R, Barreiro-de Acosta M, Lorenzo-Gonzalez A, et al. Accuracy of Consecutive Fecal Calprotectin Measurements to Predict Relapse in Inflammatory Bowel Disease Patients Under Maintenance With Anti-TNF Therapy: A Prospective Longitudinal Cohort Study. *J Clin Gastroenterol* 2018;52(3):229-34.
48. Atalay S, van den Reek J, van Vugt LJ, et al. Tight controlled dose reduction of biologics in psoriasis patients with low disease activity: a randomized pragmatic non-inferiority trial. *BMC dermatology* 2017;17(1):6. doi: 10.1186/s12895-017-0057-6 [published Online First: 2017/05/10]

For peer review only

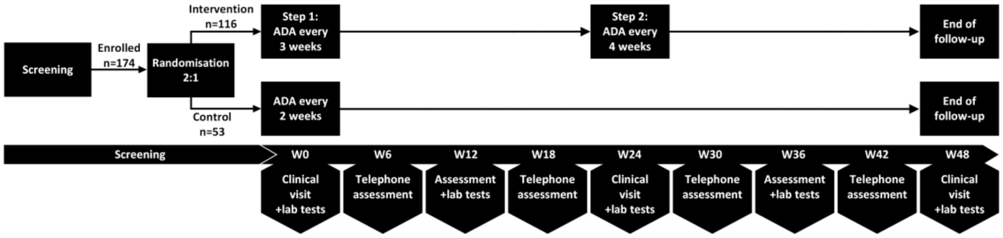

Schematic presentation of the trial design.
ADA = adalimumab, W0 = week 0, W6 = week 6, etc. Lab tests include hemoglobin, leukocytes, thrombocytes, albumin, C-reactive protein, calprotectin

281x66mm (300 x 300 DPI)

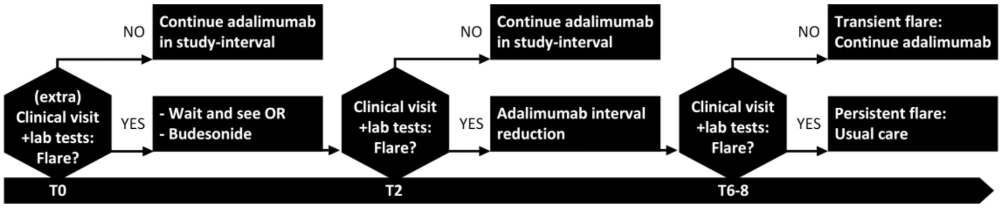

Protocolised treatment recommendation in case of disease flare
T0 = start of possible disease flare, which can occur at any time during follow-up, T2 = 2 weeks after T0, T6-8 = 6-8 weeks after T0. Lab tests include hemoglobin, leukocytes, thrombocytes, albumin, C-reactive protein, calprotectin

283x57mm (300 x 300 DPI)

Supplementary table 1: SPIRIT checklist

Section/item	Item No	Description	Addressed on page number
**Administrative information**			
Title	1	Descriptive title identifying the study design, population, interventions, and, if applicable, trial acronym	1
Trial registration	2a	Trial identifier and registry name. If not yet registered, name of intended registry	8, abstract
	2b	All items from the World Health Organization Trial Registration Data Set	Throughout entire protocol
Protocol version	3	Date and version identifier	8
Funding	4	Sources and types of financial, material, and other support	19
Roles and responsibilities	5a	Names, affiliations, and roles of protocol contributors	20
	5b	Name and contact information for the trial sponsor	20
	5c	Role of study sponsor and funders, if any, in study design; collection, management, analysis, and interpretation of data; writing of the report; and the decision to submit the report for publication, including whether they will have ultimate authority over any of these activities	20
	5d	Composition, roles, and responsibilities of the coordinating centre, steering committee, endpoint adjudication committee, data management team, and other individuals or groups overseeing the trial, if applicable (see Item 21a for data monitoring committee)	20-22
**Introduction**			
Background and rationale	6a	Description of research question and justification for undertaking the trial, including summary of relevant studies (published and unpublished) examining benefits and harms for each intervention	4-5
	6b	Explanation for choice of comparators	4-5
Objectives	7	Specific objectives or hypotheses	5-6

For peer review only

Trial design	8	Description of trial design including type of trial (eg, parallel group, crossover, factorial, single group), allocation ratio, and framework (eg, superiority, equivalence, noninferiority, exploratory)	8
**Methods: Participants, interventions, and outcomes**			
Study setting	9	Description of study settings (eg, community clinic, academic hospital) and list of countries where data will be collected. Reference to where list of study sites can be obtained	8, 21-22
Eligibility criteria	10	Inclusion and exclusion criteria for participants. If applicable, eligibility criteria for study centres and individuals who will perform the interventions (eg, surgeons, psychotherapists)	9
Interventions	11a	Interventions for each group with sufficient detail to allow replication, including how and when they will be administered	8-10, Figure 1, Table 1
	11b	Criteria for discontinuing or modifying allocated interventions for a given trial participant (eg, drug dose change in response to harms, participant request, or improving/worsening disease)	10, Figure 2
	11c	Strategies to improve adherence to intervention protocols, and any procedures for monitoring adherence (eg, drug tablet return, laboratory tests)	11
	11d	Relevant concomitant care and interventions that are permitted or prohibited during the trial	9-10
Outcomes	12	Primary, secondary, and other outcomes, including the specific measurement variable (eg, systolic blood pressure), analysis metric (eg, change from baseline, final value, time to event), method of aggregation (eg, median, proportion), and time point for each outcome. Explanation of the clinical relevance of chosen efficacy and harm outcomes is strongly recommended	6-7, 9-12
Participant timeline	13	Time schedule of enrolment, interventions (including any run-ins and washouts), assessments, and visits for participants. A schematic diagram is highly recommended (see Figure)	Figure 1
Sample size	14	Estimated number of participants needed to achieve study objectives and how it was determined, including clinical and statistical assumptions supporting any sample size calculations	13
Recruitment	15	Strategies for achieving adequate participant enrolment to reach target sample size	8
**Methods: Assignment of interventions (for controlled trials)**			

Allocation:			
Sequence generation	16a	Method of generating the allocation sequence (eg, computer-generated random numbers), and list of any factors for stratification. To reduce predictability of a random sequence, details of any planned restriction (eg, blocking) should be provided in a separate document that is unavailable to those who enrol participants or assign interventions	13
Allocation concealment mechanism	16b	Mechanism of implementing the allocation sequence (eg, central telephone; sequentially numbered, opaque, sealed envelopes), describing any steps to conceal the sequence until interventions are assigned	13
Implementation	16c	Who will generate the allocation sequence, who will enrol participants, and who will assign participants to interventions	13
Blinding (masking)	17a	Who will be blinded after assignment to interventions (eg, trial participants, care providers, outcome assessors, data analysts), and how	13
	17b	If blinded, circumstances under which unblinding is permissible, and procedure for revealing a participant's allocated intervention during the trial	Not applicable: unblinded
**Methods: Data collection, management, and analysis**			
Data collection methods	18a	Plans for assessment and collection of outcome, baseline, and other trial data, including any related processes to promote data quality (eg, duplicate measurements, training of assessors) and a description of study instruments (eg, questionnaires, laboratory tests) along with their reliability and validity, if known. Reference to where data collection forms can be found, if not in the protocol	9-11
	18b	Plans to promote participant retention and complete follow-up, including list of any outcome data to be collected for participants who discontinue or deviate from intervention protocols	-
Data management	19	Plans for data entry, coding, security, and storage, including any related processes to promote data quality (eg, double data entry; range checks for data values). Reference to where details of data management procedures can be found, if not in the protocol	8
Statistical methods	20a	Statistical methods for analysing primary and secondary outcomes. Reference to where other details of the statistical analysis plan can be found, if not in the protocol	14-15

	20b	Methods for any additional analyses (eg, subgroup and adjusted analyses)	14-15
	20c	Definition of analysis population relating to protocol non-adherence (eg, as randomised analysis), and any statistical methods to handle missing data (eg, multiple imputation)	-
**Methods: Monitoring**			
Data monitoring	21a	Composition of data monitoring committee (DMC); summary of its role and reporting structure; statement of whether it is independent from the sponsor and competing interests; and reference to where further details about its charter can be found, if not in the protocol. Alternatively, an explanation of why a DMC is not needed	8, 22
	21b	Description of any interim analyses and stopping guidelines, including who will have access to these interim results and make the final decision to terminate the trial	Not applicable
Harms	22	Plans for collecting, assessing, reporting, and managing solicited and spontaneously reported adverse events and other unintended effects of trial interventions or trial conduct	10
Auditing	23	Frequency and procedures for auditing trial conduct, if any, and whether the process will be independent from investigators and the sponsor	Not applicable
**Ethics and dissemination**			
Research ethics approval	24	Plans for seeking research ethics committee/institutional review board (REC/IRB) approval	8
Protocol amendments	25	Plans for communicating important protocol modifications (eg, changes to eligibility criteria, outcomes, analyses) to relevant parties (eg, investigators, REC/IRBs, trial participants, trial registries, journals, regulators)	8 standard according to Dutch Medical Research Involving Human Subjects Act (WMO)
Consent or assent	26a	Who will obtain informed consent or assent from potential trial participants or authorised surrogates, and how (see Item 32)	8 standard according to Dutch Medical Research Involving Human Subjects Act (WMO)
	26b	Additional consent provisions for collection and use of participant data and biological specimens in ancillary studies, if applicable	Not applicable

Confidentiality	27	How personal information about potential and enrolled participants will be collected, shared, and maintained in order to protect confidentiality before, during, and after the trial	8
Declaration of interests	28	Financial and other competing interests for principal investigators for the overall trial and each study site	20
Access to data	29	Statement of who will have access to the final trial dataset, and disclosure of contractual agreements that limit such access for investigators	-
Ancillary and post-trial care	30	Provisions, if any, for ancillary and post-trial care, and for compensation to those who suffer harm from trial participation	-
Dissemination policy	31a	Plans for investigators and sponsor to communicate trial results to participants, healthcare professionals, the public, and other relevant groups (eg, via publication, reporting in results databases, or other data sharing arrangements), including any publication restrictions	No publication restrictions
	31b	Authorship eligibility guidelines and any intended use of professional writers	Not applicalble
	31c	Plans, if any, for granting public access to the full protocol, participant-level dataset, and statistical code	-
**Appendices**			
Informed consent materials	32	Model consent form and other related documentation given to participants and authorised surrogates	-
Biological specimens	33	Plans for collection, laboratory evaluation, and storage of biological specimens for genetic or molecular analysis in the current trial and for future use in ancillary studies, if applicable	Not applicable

For peer review only

BMJ Open

Lengthening Adalimumab Dosing Interval in quiescent Crohn's disease patients: protocol for the pragmatic randomised non-inferiority LADI study.

Journal:	*BMJ Open*
Manuscript ID	bmjopen-2019-035326.R1
Article Type:	Protocol
Date Submitted by the Author:	03-Feb-2020
Complete List of Authors:	Smits, Lisa; Radboudumc, Department of Gastroenterology and Hepatology<br>Pauwels, Renske Wilhelmina Maria; Erasmus Medical Center, Department of Gastroenterology and Hepatology<br>Kievit, Wietske; Radboudumc, Department for Health Evidence<br>Jong, D; Radboudumc, Department of Gastroenterology and Hepatology<br>De Vries, Annemarie; Erasmus Medical Center, Department of Gastroenterology and Hepatology<br>hoentjen, F; Radboudumc, Department of Gastroenterology and Hepatology<br>van der Woude, Janneke; Erasmus Medical Center, Department of Gastroenterology and Hepatology
**Primary Subject Heading**:	Gastroenterology and hepatology
Secondary Subject Heading:	Evidence based practice, Research methods
Keywords:	Inflammatory bowel disease < GASTROENTEROLOGY, Gastroenterology < INTERNAL MEDICINE, Adverse events < THERAPEUTICS, Clinical trials < THERAPEUTICS, HEALTH ECONOMICS, STATISTICS & RESEARCH METHODS

SCHOLARONE™
Manuscripts

I, the Submitting Author has the right to grant and does grant on behalf of all authors of the Work (as defined in the below author licence), an exclusive licence and/or a non-exclusive licence for contributions from authors who are: i) UK Crown employees; ii) where BMJ has agreed a CC-BY licence shall apply, and/or iii) in accordance with the terms applicable for US Federal Government officers or employees acting as part of their official duties; on a worldwide, perpetual, irrevocable, royalty-free basis to BMJ Publishing Group Ltd ("BMJ") its licensees and where the relevant Journal is co-owned by BMJ to the co-owners of the Journal, to publish the Work in this journal and any other BMJ products and to exploit all rights, as set out in our licence.

The Submitting Author accepts and understands that any supply made under these terms is made by BMJ to the Submitting Author unless you are acting as an employee on behalf of your employer or a postgraduate student of an affiliated institution which is paying any applicable article publishing charge ("APC") for Open Access articles. Where the Submitting Author wishes to make the Work available on an Open Access basis (and intends to pay the relevant APC), the terms of reuse of such Open Access shall be governed by a Creative Commons licence – details of these licences and which Creative Commons licence will apply to this Work are set out in our licence referred to above.

Other than as permitted in any relevant BMJ Author's Self Archiving Policies, I confirm this Work has not been accepted for publication elsewhere, is not being considered for publication elsewhere and does not duplicate material already published. I confirm all authors consent to publication of this Work and authorise the granting of this licence.

For peer review only

Lengthening Adalimumab Dosing Interval in quiescent Crohn's disease patients: protocol for the pragmatic randomised non-inferiority LADI study.

L.J.T. Smits[1*], R.W.M. Pauwels[2*], W. Kievit[3], D.J. de Jong[1], A.C. de Vries[2], F. Hoentjen[1#], C.J. van der Woude[2#]

* Authors share first authorship
\# Authors share last authorship
1. Radboud UMC, Department of Gastroenterology and Hepatology, Nijmegen, The Netherlands
2. Erasmus MC, Department of Gastroenterology and Hepatology, Rotterdam, The Netherlands
3. Radboud UMC, Department for Health Evidence, Nijmegen, The Netherlands

Short title: Adalimumab tapering in Crohn's disease

Correspondence
C.J. van der Woude
e-mail: c.vanderwoude@erasmusmc.nl
telephone: 0031 10 703 3020
Erasmus MC, Department of Gastroenterology and Hepatology
P.O. Box 2040
3000 CA Rotterdam
The Netherlands

Word count

4503

For peer review only

ABSTRACT

Introduction: Adalimumab is effective for maintenance of remission in patients with Crohn's disease (CD) at a dose of 40mg subcutaneously every 2 weeks. However, adalimumab is associated with (long-term) adverse events and is costly. Cohort studies showed that interval lengthening may be a successful treatment strategy in a significant proportion of CD patients. The aim of this study is to demonstrate non-inferiority and cost-effectiveness of disease activity guided adalimumab interval lengthening compared to standard dosing of every other week (EOW).

Methods and analysis: The LADI study (Lengthening Adalimumab Dosing Interval) is a pragmatic, multicentre, open label, randomised controlled non-inferiority trial. 174 CD patients on adalimumab maintenance therapy in long-term (> 9 months) clinical and biochemical remission will be included (C-reactive protein (CRP) ≤10 mg/L, fecal calprotectin (FC) ≤150 mg/kg, Harvey-Bradshaw Index (HBI) <5). Patients will be randomised 2:1 into the intervention (adalimumab interval lengthening) or control group (adalimumab EOW). The intervention group will lengthen the adalimumab administration interval to every 3 weeks, and after 24 weeks to every 4 weeks. Clinical and biochemical disease activity is monitored every 12 weeks by physician global assessment, HBI, CRP and FC. In case of disease flare, dosing will be increased.

Primary outcome: Non-inferiority in cumulative incidence of persistent (>8 weeks) disease flares in 48 weeks of follow-up. A flare is defined as two of three of the following criteria; FC >250 µg/g, CRP≥10 mg/L, HBI ≥5. Non-inferiority margin is 15%. Secondary outcomes include cumulative incidence of transient flares, adverse events, predictors for successful dose reduction and cost-effectiveness.

Ethics and dissemination: The study is approved by the Medical Ethics Committee Arnhem-Nijmegen, the Netherlands (registration number NL58948.091.16). Results will be published in peer-reviewed journals and presented at international conferences.
Trial registration: EudraCT: 2016-003321-42. Registered on 26 September 2016.
Clinicaltrials.gov: NCT03172377. Registered on 1 June 2017.

Keywords
Crohn's disease; anti-TNF; adalimumab; calprotectin; dose reduction; interval lengthening; non-inferiority; cost-effectiveness; inflammatory bowel disease

For peer review only

STRENGTHS AND LIMITATIONS OF THIS STUDY

- The LADI study is the first randomised controlled trial that investigates adalimumab interval lengthening in Crohn's disease patients
- This pragmatic study is clinically relevant and results can easily be implemented in daily practice
- The National Crohn and colitis patients organisation is involved and patient-reported outcomes are included
- The study is not blinded

For peer review only

BACKGROUND

Crohn's disease (CD) is a chronic inflammatory disease of the gastrointestinal tract, characterized by a relapsing and remitting disease course. Patients show an abnormal mucosal immune response, resulting from an interplay of genetic susceptibility, environmental factors and the intestinal microflora.[1] Treatment consists of immunosuppressive medication, including monoclonal antibodies that block tumor necrosis factor alpha (anti-TNF); such as infliximab, adalimumab and certolizumab. Adalimumab is a humanised anti-TNF antibody that is effective as both induction and maintenance therapy for CD.[2-4] Adalimumab is administered by subcutaneous (sc) injection and an induction dose of 80 mg (week 0) and 40 mg (week 2) or 160mg (week 0) and 80mg (week 2) are generally used, followed by 40mg every 2 weeks.[5]

Although adalimumab is generally safe, side effects do occur. The risk of (opportunistic) infections is increased, especially in combination with immunosuppressive therapies, most often thiopurines or methotrexate.[6-9] A recently published review on long term safety of adalimumab (n=3606 CD patients) showed a high absolute risk of any infection of 119 events per 100 patient years (PYs) and a risk of serious infection of 6.7/100 PYs in this selected trial-population with relatively low comorbidity.[10] The incidence rate of injection site reactions (ISR: local pain and swelling) was 7.7/100 PYs).[10] In addition, several reports show an increased risk of skin cancer (both melanoma and non-melanoma skin cancer), especially in combination with thiopurines.[6 7 9 11 12] In addition to potential side effects, the costs of adalimumab are significant. Before the introduction of biosimilars, the costs of anti-TNF in the Netherlands were €15.000 – 30.000 per CD patient annually.[13 14] Anti-TNF including adalimumab is expected to continue to be the main cost driver of CD management for several reasons. First, the number of CD patients is increasing in the Netherlands.[15] Secondly, recent data stimulate an early use of anti-TNF with an accelerated step-up or top-down approach in combination with treat-to-target (mucosal healing), to prevent bowel damage.[16] Thirdly, the entry of lower cost biosimilars will possibly cause physicians to preferentially prescribe anti-TNF treatment, which will increase its use.[17-19]

Discontinuation of adalimumab therapy in CD patients in stable clinical remission is a clinical strategy that may aid in reducing the risk of side effects, costs, and avoid prolonged immunosuppression during a quiescent disease course. However, in a large meta-analysis on individual patient data (n=1264, i.e. including the landmark study by Louis et al.[20]) on cessation of anti-TNF therapy, approximately 37% of the patients had a relapse in one year, and 52% after two years of follow-up (Pauwels et al., unpublished data). Therefore, an alternative strategy of dose reduction of adalimumab rather than discontinuation may be

For peer review only

considered. In RA, the DRESS study concluded that disease activity guided dose reduction of anti-TNF is non-inferior and cost-effective, compared to maintaining regular dosing.[21 22] However, extrapolation of these results to CD is questionable, since RA patients generally use different concomitant medication, suffer from different comorbidities and anti-TNF shows different pharmacodynamic characteristics in RA patients.[23 24] In CD, adalimumab dose reduction is uncommon in daily practice. Only two retrospective cohort studies (n=46+40) reported CD patients who used adalimumab 40mg every three weeks (ETW).[25 26] After a median follow-up of 16 and 24 months, respectively 63% and 65% remained in clinical remission.

The aim of this randomised controlled trial is to demonstrate non-inferiority and cost-effectiveness of disease activity guided adalimumab injection interval lengthening compared to standard of care (continued EOW dosing) in maintaining remission in CD. In this paper we describe the study design as well as potential pitfalls and outcomes.

For peer review only

OBJECTIVE

Primary objective

- To demonstrate non-inferiority of disease activity guided adalimumab injection interval lengthening compared to adalimumab EOW dosing (standard of care) in CD patients in stable disease remission at 48 weeks of follow-up. Non-inferiority is reached if the difference in cumulative incidence of persistent flares not exceeds the non-inferiority margin. A persistent flare is defined as two of three of the following criteria, persisting for > 8 weeks despite dose escalation of adalimumab:
  - Fecal calprotectin (FC) >250 µg/g
  - C-reactive protein (CRP) ≥10 mg/L
  - Harvey-Bradshaw Index (HBI) ≥5

Secondary objectives

- To report the proportion of patients that had successful interval lengthening, defined as the absence of a disease flare, while treated with adalimumab ETW or every four weeks (EFW), at week 48.
- To identify factors that are associated with successful interval lengthening (e.g. baseline patient and treatment characteristics, FC, CRP, adalimumab drug levels and antibodies to adalimumab).
- To compare the cumulative incidence of patients with a transient flare (duration ≤8 weeks) between the intervention and control group at week 48.
- To compare the proportion of patients that used budesonide, prednisone or other immunomodulators in order to treat a (transient) flare.
- To compare the proportion of patients in clinical and biochemical remission between the intervention and control group at week 48. Remission is defined as a HBI <5, FC <150 µg/g and CRP <10 mg/L. In case disease activity is assessed with endoscopy or magnetic-resonance-imaging (MRI) scan, that conclusion overrules our definition.
- To compare inflammatory bowel disease (IBD)-specific quality of life by the short-IBD questionnaire (SIBDQ)) between the intervention and control group every 12 weeks during follow-up.
- To compare disease activity by HBI and patient reported outcome (PRO-2) between the intervention and control group every 12 weeks during follow-up.
- To compare medical consumption (by iMTA MCQ questionnaire) and work productivity (by iMTA PCQ questionnaire) between the intervention and control group until week 48, in order to calculate the decremental cost effectiveness ratio of this interval lengthening strategy.

For peer review only

- To compare the rates of (serious) adverse events ((S)AEs) that are (possibly) related to adalimumab and the rates of (S)AEs that are (possibly) related to adalimumab interval lengthening between the intervention and control group, expressed as events/ 100 PYs of follow-up.
- To compare adalimumab use between the intervention and control group, including the cumulative dose during follow-up, the proportion of patients that uses adalimumab ETW and EFW.

For peer review only

METHODS

This protocol includes the standard protocol items recommended for interventional trials according to the SPIRIT guidelines (Supplementary file 1).[27]

Design

This randomised controlled trial is currently being performed at the departments of Gastroenterology and Hepatology in 23 hospitals in the Netherlands, including both academic and non-academic centres. The aim of the adalimumab interval lengthening strategy is to minimize the amount of adalimumab use while maintaining remission in CD. Therefore, longer adalimumab intervals will be compared with adalimumab EOW in a non-inferiority design (to show the same effect is maintained with a dose reduction strategy), instead of a superiority design, which is used to demonstrate that an intervention leads to superior outcomes than the standard of care. The rationale behind a non-inferiority design is that benefits may be present in other areas (i.e. fewer side effects, lower costs) so that the intervention would be preferred if its efficacy is not worse.

The date of the first enrollment was 3 May 2017. The study is approved by the Medical Ethical Committee (METC) Arnhem-Nijmegen (registration number NL58948.091.16). Important protocol modifications are assessed and approved by the METC, and reported to participating investigators. The most recent study protocol version 3.3 (July 2018) is presented in this manuscript. The LADI study has been registered at clinicaltrials.gov (NCT03172377) and the Dutch trial register (NTRID6417). A data safety monitoring board (DSMB) is installed in order to independently assess the efficacy and safety of the study intervention and to monitor the overall conduct of the trial. Prior to enrollment, all patients have to sign informed consent (Supplementary file 2).

Patient and Public Involvement

The study was designed in collaboration with the Dutch Crohn's and colitis patient organisation (CCUVN) in order to optimise patient participation. We based our study design on the results of a *biological focus group* by members of the CCUVN. This focus group showed that patients do accept a reduction of the dose of their biological agent. Additionally, based on previous interactions with the CCUVN, we have included patient focused outcomes in our study, such as the quality of life and PRO-2.

For peer review only

In- and exclusion criteria

All adult CD patients with colonic and/or distal ileal and/or proximal CD, who are treated with adalimumab 40 mg every 2 weeks at a stable dose, at least 9 months in steroid-free clinical remission and not scheduled for CD-related surgery, are eligible for participation.[28] Remission is defined as a HBI <5, FC <150 µg/g and CRP <10 mg/L. The current guidelines from the European Crohn's and Colitis Organisation suggest to use CRP <10 mg/L for the definition of disease remission [5]. Endoscopic assessment prior to enrollment is not mandatory, however if an ileocolonoscopy was performed before the start of the study and demonstrated complete mucosal healing (Simple Endoscopic Score-CD <3 or no ulcerations), a FC<250 µg/g is accepted as inclusion criterium. Permitted concomitant CD therapies are: aminosalicylates, azathioprine, 6-mercatopurine, methotrexate and thioguanine at a stable dose for 12 weeks. Patients with arthralgia will be included, however inflammatory arthritis is an exclusion criterium, as this can provide elevated inflammatory markers. Furthermore, patients with active draining fistulas are excluded. Other exclusion criteria are pregnancy or lactation and other significant medical conditions that might interfere with this study (such as a current/recent malignancy, immunodeficiency syndromes and psychiatric illness), or when it is to be expected that the outcome cannot be measured (short life expectancy, planned major surgery, language issues).

Study groups

Control group

The control group continues the maintenance adalimumab sc treatment 40mg EOW. Treatment decisions are made at the discretion of the treating physician. Of note, dose reduction beyond 40 mg per two weeks is currently not recommended according to national guidelines.[29] Patients follow a standardized protocol based on the tight control/treat-to-target principle in order to maintain low disease activity.[16]

Intervention group

Adalimumab interval will be lengthened through a stepwise disease activity guided manner.

Step 1: Upon inclusion, the interval will be prolonged to ETW.

Step 2: After week 24, patients in remission will lengthen their dosing interval to EFW.

Step 3: If adalimumab interval lengthening leads to a confirmed flare, patients will return to the preceding effective interval (Figure 1). If a flare is not objectively confirmed, patients are advised to continue adalimumab in their study-interval. However, interval reduction is accepted if patients really want this as this situation reflects daily clinical practice.

For peer review only - http://bmjopen.bmj.com/site/about/guidelines.xhtml

In contrast to the DRESS study, the discontinuation of therapy after successful de-escalation to 40 mg EFW is not implemented in the study protocol.[21] Total follow-up time will be 48 weeks. Follow-up visits and outcome measurements are similar to the control group.

Co-intervention

The use of previously mentioned concomitant medication is allowed and must be documented on the CRF (stating type, dosage and duration). If possible, existing concomitant medication should not be changed during the study.

If patients experience worsening of symptoms in between visits, they must contact the outpatient clinic. For further treatment of the flare, patients in the control arm are referred to their treating physician. In the intervention arm, patients will return to the preceding effective adalimumab dosing interval (Figure 1). The decision to start concomitant therapy remains at the discretion of the treating physician.

Secondary outcome measurements

- Quality of life

For assessment of quality of life, we will use the short IBDQ, which is a validated and disease-specific questionnaire.[30]

- Patient reported disease activity

We will use the only validated IBD patient-reported outcome measure, 'PRO-2', consisting of reported diarrhea and abdominal pain.[31]

- Factors associated with successful dose reduction

Factors which are possibly related to successful dose reduction include: baseline patient and treatment characteristics, adalimumab drug levels (μg/mL) and antibodies (AU/mL), clinical (physician global assessment (PGA), HBI) and laboratory results (FC (μg/g), CRP (mg/L), haemoglobin (mmol/L), leucocytes (10^9/L), platelets (10^9/L), albumin (g/L)).

- Safety

AEs and SAEs are registered during follow-up. All SAEs are reported to the METC Arnhem-Nijmegen.

- Cost-effectiveness

The impact of dose reduction on the quality of life of patients will be assessed by the EQ-5D at 24 and 48 weeks following randomisation, compared to baseline. The EQ-5D utility will be

For peer review only

used to derive a quality-adjusted life year (QALY) estimate for each patient according to the trapezium rule.[32 33]

Assessments

Enrolled patients will visit the outpatient clinic every 24 weeks. If preferred by the patient or treating physician, the evaluations at week 12 and 36 can take place as outpatient clinic visit as well. Every 12 weeks, laboratory tests (e.g. FC, CRP, haemoglobin and albumin) will be performed. At week 0, 24 and 48 serum samples are stored for measurement of adalimumab drug levels and antibodies to adalimumab. Additionally, patients in both arms will be interviewed via telephone every 6 weeks in between clinical visits to assess for adverse events, symptoms and potential disease activity. If such an interview suggests a disease flare, patients must visit the outpatient clinic in order to undergo complete disease activity assessment and laboratory and FC tests. If patients have a flare at week 48, disease activity will be monitored until disease remission, in order to define the flare as persistent- or transient flare. In addition, study questionnaires are automatically sent via Castor every 6 weeks. During follow-up, patients register the adalimumab injection dates in a study-diary and bring this to the outpatient clinic every visit to evaluate adherence to adalimumab. An overview of all visits and assessments is depicted in Table 1, Figure 1 and Figure 2.

For peer review only

Table 1. SPIRIT schedule of enrollment, interventions, and assessments

	STUDY PERIOD											
	Enrollment	Allocation	Follow-up									Extra
**TIMEPOINT**	$-t_1$	0	w_0	w_6	w_{12}	w_{18}	w_{24}	w_{30}	w_{36}	w_{42}	w_{48}	w_e
**ENROLLMENT:**												
**Eligibility screen**	X											
**Informed consent**	X											
**Allocation**		X										
**INTERVENTIONS:**												
Intervention: Lengthening adalimumab dosing interval			◆	—	—	—	—	—	—	—	◆	
Control: Adalimumab every other week			◆	—	—	—	—	—	—	—	◆	
**ASSESSMENTS:**												
Medical history	X	X										
Laboratory tests*			X		X		X		X		X	X
Fecal calprotectin			X		X		X		X		X	X
Storage of serum samples			X				X				X	X
Concomitant medication			X	X	X	X	X	X	X	X	X	X
(Serious) adverse events			X	X	X	X	X	X	X	X	X	X
Physician global assessment			X	X	X	X	X	X	X	X	X	X
HBI and PRO-2			X		X		X		X		X	X
IBD-Q and EQ5D			X		X		X		X		X	X
iMTA MCQ, -PCQ			X		X		X		X		X	X

**Hemoglobin, leukocytes, thrombocytes, albumin, C-reactive protein.*

For peer review only

HBI = Harvey Bradshaw Index, PRO-2 = Patient Reported Outcome-2, IBD-Q = Inflammatory Bowel Disease Questionnaire, EQ5D = EuroQuol 5D, iMTA MCQ = institute for Medical Technology Assessment Medical Consumption Questionnaire, PCQ = Productivity Cost Questionnaire

Randomisation, allocation concealment, stratification

Patients are randomised by the research physician using a computer-generated randomisation system (Castor). Castor uses a validated variable block randomisation model with block sizes of 6, 9 and 12. Patients will be randomised in a 2:1 ratio for the intervention or the control group, respectively. We chose 2:1 randomisation to stimulate patient inclusion, as patients have a higher chance to randomise for the intervention group. Furthermore, more determinants can be included in a prediction model for successful dose reduction.

Patients will be stratified on co-medication use (yes/no), as the incidence of flares could possibly be different with or without co-medication use. Co-medication includes azathioprine, 6-mercaptopurine, 6-thioguanine, methotrexate. Both patients and physicians are un blinded, as we aim to represent daily practice during this pragmatic study .

Sample size

The null hypothesis in non-inferiority studies is that the intervention is inferior compared to the control arm by more than the non-inferiority margin. The alternative hypothesis is that the intervention is not worse than the control by more than the non-inferiority margin. Therefore, if the null hypothesis is rejected, the alternative hypothesis that the intervention is non-inferior is accepted.[34] Based on an extrapolation of data from the DRESS study and results from a real-life CD cohort in Leuven, an estimated 15% of patients will experience the primary outcome (persistent flare) in the control arm. In the Leuven cohort, 41/156 (26%) patients discontinued adalimumab due to loss of response, despite adalimumab dose escalation.[21 35] The latter 26% was adjusted to an expected 15% for our cohort because the follow-up time in our cohort concerns 12 rather than 20 months, and our cohort is a preselected cohort of patients in long and stable remission rather than a cross-sectional cohort. In non-inferiority analyses, one-sided testing is used. Applying one sided testing, an alpha of 0.05 ($Z\alpha = 1.64$), power 1-beta 0.8 ($Z\beta = 0.84$), an non-inferiority margin of 15% and randomisation ratio of 2:1 intervention versus control resulted in n = 105 and n = 53 for intervention and control arm, respectively. Accounting for a 10% drop-out, 174 patients have to be included in total.

A non-inferiority margin of 15% means a maximum difference in persistent flare of 15% between the usual care and intervention group. We believe this strikes an acceptable balance between the potential harms of flare, and the benefits of dose reduction (fewer injections, potential for reduced risk of side effects and cost-savings). The large Nor-Switch

For peer review only

trial also used a non-inferiority margin of 15% for disease worsening during follow-up.[36] Based on this example, discussions in our study-group and approval of the protocol by the Dutch Organisation for Health Research and Development, we believe this margin is appropriate. The DRESS study used a non-inferiority margin of 20%. Although side effects/SAEs of adalimumab seem comparable in RA versus IBD, rheumatologists probably accept a higher proportion of flares because there are more alternative biological therapies available, thus a loss of effect of one biological therapy might be given less weight in RA.[37]

Planned data analysis

The primary outcome; cumulative incidence of persistent flares will be expressed as proportions in both groups. A confidence interval for the difference between study groups will be determined (adjusted for co-medication use at baseline using the Cochran-Mantel-Haenszel procedure, as this variable is used for stratification in the randomisation process[38]). The upper limit of the confidence interval will be compared with the non-inferiority margin. We will use both intention to treat and per protocol analyses, as the latter is considered the most conservative analysis for non-inferiority trials.[39] Patients in the interval lengthening group are included in the per protocol analyses if they: lengthened the adalimumab interval at least to three weeks, regardless whether they returned to a preceding effective interval in case of a disease flare. Patients in the control group are included in the per protocol analyses if they: used adalimumab EOW without consistent interval lengthening, incidental postponement of an injection during infection or around holidays is allowed. Descriptive patient (and treatment) baseline variables will be summarized as means ± SD, medians with interquartile ranges or percentages, depending on the type of measurement. Gender, BMI, age, prior medication for CD, disease duration, Montreal classification, IBD-related surgical history, comorbidity, inflammatory parameters including HBI, FC, CRP, adalimumab drug levels and antibodies to adalimumab will be reported.

The secondary continuous outcomes HBI, SIBDQ, PRO-2, adalimumab drug levels and antibody levels at 48 weeks will be analysed by either student's t-test or Mann Whitney U test depending on the type of distribution of the data. In addition, the course over time for several continuous outcomes measured at multiple time points (every 12 weeks) will be analysed using repeated measures analyses in which the outcome can be corrected for the baseline value of the specific outcome and potential confounding factors. The number of (S)AEs that are (possibly) related to adalimumab or to adalimumab interval lengthening will be reported as rates, defined as events/100 PYs of follow-up; details of these (S)AEs will be provided. In the intervention group, patient characteristics and clinical features will be analysed to predict a persistent flare. A prediction model will be developed and fitted using a univariable

For peer review only - http://bmjopen.bmj.com/site/about/guidelines.xhtml

selection based on a p-value <0.2 and a multivariable approach with backward selection. Predictive accuracy will be determined by the area under the receiver operating curve. A two-sided P-value of <0.05 is considered statistically significant. All statistical analyses will be performed by using IBM SPS Statistics 25.0.

Data analysis: Cost effectiveness

The cost analysis consists of two main parts. First, at patient level, volumes of care related to the CD care and anti-TNF therapy will be measured by means of the iMTA Medical Consumption Questionnaire (MCS). This questionnaire measures all relevant health care related costs like outpatient visits at any medical specialist, hospitalizations and imaging procedures. Loss of productivity due to illness or recovery in patients below the age of 65 will be estimated based on patient reported absences from paid (or unpaid) labor measured with the Productivity Cost Questionnaire (PCS). The second part of the cost analysis consists of determining the cost prices for each volume of consumption. The standard cost prices from the 'Dutch Guidelines for Cost Analyses' and www.medicijnkosten.nl will be used. For units of care where no standard prices are available real costs prices will be determined on the basis full cost pricing. Productivity losses will be valued by means of the friction cost method. In the end volumes of care will be multiplied with the cost prices for each volume of care to calculate costs. Because we anticipate non-inferiority of the dose reduction strategy we will primarily analyse cost-savings: direct medical cost as well as total costs (medical and non-medical costs) will be compared between intervention and control group. A possible small but acceptable loss of effect can be incorporated in the analyses by determining a decremental cost-effectiveness ratio (DCER) by dividing the difference in costs by the difference in QALYs between the groups. The DCER expresses with how much money a loss of 1 QALY is compensated. If this amount is high the decision makers may be willing to accept a loss of effect. Uncertainty in the DCER will be non-parametrically determined using bootstrap techniques (1000 replications). Results from this analysis will be presented in a scatter plot and willingness to pay (or accept) curve. Furthermore the Net Monetary Benefit (NMB) per patient will be calculated for different levels of willingness to accept (WTA) in euro's per QALY, using the formula: WTA * effect (difference in QALY) - costs. This results in the net amount of money saved, when the possible loss of QALY is corrected for, using different WTA levels per QALY.

Ethics and dissemination

The study is approved by the Medical Ethics Committee Arnhem-Nijmegen, the Netherlands (registration number NL58948.091.16). Data of all participating centres will be collected by electronic case-report forms (CRF's) and monitored following good clinical practice (GCP)

For peer review only - http://bmjopen.bmj.com/site/about/guidelines.xhtml

guidelines. The collected data will be entered in Castor, an electronic database set up for clinical trials (https://www.castoredc.com). Data will be coded and kept based on the rules for GCP by certified personnel. Results will be published in peer-reviewed journals and presented at international conferences.

For peer review only

DISCUSSION

Dose reduction of adalimumab in CD patients with stable disease may provide similar disease control but reduction of adverse events and costs. With this pragmatic, non-inferiority study design we aim to evaluate the outcomes of this strategy. Only two small retrospective studies reported on adalimumab 40mg ETW in CD patients.[25 26] No prospective randomised data are available. Prior studies have investigated the effect of discontinuation of anti-TNF therapy in CD.[40-43] Previous clinical trials on withdrawal of anti-TNF after a period of prolonged remission in CD patients showed a relatively consistent profile of 42% relapses after anti-TNF cessation within one year of follow-up.[40-43] Louis et al. identified risk factors for disease flare after discontinuation of infliximab in CD patients who used infliximab and thiopurine combination therapy for at least one year. Risk factors for relapse included male sex, high leukocyte counts, high CRP, high FC and low levels of hemoglobin.[20] The multicentre randomised CEASE trial (ZonMw project number 848101009) will further investigate cessation of anti-TNF. As cessation of anti-TNF therapy is a different research question with different outcome measures, uncertainty remains on factors that are associated with successful adalimumab interval lengthening and the LADI study will provide useful information for daily clinical practice.

We decided to assess non-inferiority with regard to persistent flares (persisting >8 weeks independent of treatment changes such as adalimumab dose escalation) since these are the most relevant clinical outcomes in this setting. Temporary flares (persisting <8 weeks) that resolve after appropriate treatment are less difficult to manage and are likely to occur as an acceptable result of searching for the optimal individualized treatment interval. Temporary flares will still function as relevant secondary outcome in our trial. For the definition of a flare, 2 consecutive measurements demonstrating two out of three of the following criteria; FC >250 µg/g, CRP ≥10 mg/L, HBI ≥5 are required. As it has been shown that flares are frequently temporary and occur and sometimes disappear without regimen change, a flare is only considered a flare if it is confirmed two times. For this composite endpoint we preferred to incorporate the HBI instead of the Crohn's Disease Activity Index on account of accessible clinical implementation in daily practice. In addition, FC and CRP are non-invasive, cheap and widely available biomarkers of disease activity.[44] Furthermore, FC correlates to endoscopic disease activity.[45 46] Recently, it was shown that an increase in FC can precede on the onset of clinical symptoms.[47] Indeed, due to our definition of a flare, patients without clinical symptoms can also fulfill the definition of a (biochemical) flare. In addition, the requirement of an elevation in inflammatory markers at two time points allows for the exclusion of confounders such as Clostridium difficile infection and use of NSAIDs).

For peer review only - http://bmjopen.bmj.com/site/about/guidelines.xhtml

We decided not to include endoscopy outcomes in the inclusion criteria or primary endpoint. An endoscopic procedure is a burden for patients due to the invasive procedure and the intensive preparation. In addition, we aimed for study results that may be easily implemented in daily practice. Instead of an endoscopy, we used a combination of surrogate markers of inflammation including HBI, CRP and FC to determine clinical remission. A protocolized treatment is advised when a flare occurs (Figure 1). However, treatment choices are not mandatory and bridging therapy (including steroids) is left to the discretion of the treating physician.

For the study design, a blinded design was considered, but the development, costs and administration of placebo injections would create a formidable barrier for the study. Furthermore, an un-blinded (pragmatic) design fits best with the current ideas about the external validity of cost-effectiveness studies. This design mirrors the real-life setting which is also not blinded, with respect to costs and effects. In general, an unblinded study design could result in information and attribution bias, e.g. flares in patients in whom the dose is reduced would possible be reported sooner. Because this will not lead to an underestimation of the drawbacks of a dose reduction strategy, this bias was accepted.

Our trial will provide important insights in addition to the risk of recurrence as well as the risk of persistent flares. For example, we will collect valuable series of drug measurements of adalimumab. Although the DRESS study did not show predictive value of drug levels for the success of dose reduction, daily IBD practice does apply measurement of drug levels. It is possible that drug levels at baseline, either low or high, may predict successful dose reduction.

From a societal perspective, it is important to improve the cost-effectiveness of IBD healthcare. Patients with chronic inflammatory diseases use expensive medication for many years and there is a growing amount of new (expensive) drugs that will soon be implemented in daily clinical care. In RA and psoriasis, dose reduction trials in adalimumab treated patients are performed and in RA the feasibility of this strategy was already demonstrated and results from a Dutch nation-wide psoriasis trial will follow soon.[21 48] The recent introduction of biosimilars of adalimumab will further aid in cost reduction but the new costs of this therapy will still remain significant. Therefore, cost savings due to dose reduction will remain relevant.

For peer review only

In conclusion, we designed a pragmatic randomized controlled trial to assess the non-inferiority of a strategy of adalimumab dose reduction in CD patients. Accurate prediction of successful tapering may aid in reduction of costs and adverse events to further improve care for CD patients.

For peer review only

Abbreviations

CD	Crohn's disease
HBI	Harvey-Bradshaw Index
CRP	c-reactive protein
FC	fecal calprotectin
ADA	antidrug antibodies
RA	rheumatoid arthritis
EOW	every other week
ETW	every three weeks
EFW	every four weeks
(S)AE	(serious) adverse event
PYs	patient years
IBD	inflammatory bowel disease
SIBDQ	short-IBD questionnaire
PRO-2	patient reported outcome-2
MCQ	medical consumption questionnaire
PCQ	productivity cost questionnaire
PGA	physician global assessment
TNF	tumor necrosis factor
METC	Medical Ethical Committee
DSMB	data safety monitoring board
CRF	case-report form
GCP	good clinical practice
QALY	quality-adjusted life year
DCER	decremental cost-effectiveness ratio
NMB	Net Monetary Benefit
WTA	willingness to accept

Acknowledgements

This study is supported by the Dutch Initiative on Crohn and Colitis (ICC), a nationwide network of IBD centres that aims at initiating and facilitating IBD research. We thank dr. A.A. den Broeder, rheumatologist at St. Maartenskliniek Nijmegen, for his invaluable help in designing the study.

Funding

The investigator initiated LADI study is supported by the Netherlands Organisation for Health Research and Development (ZonMw, Health Care Efficiency program, grant number

For peer review only

848015002). ZonMw is part of the Netherlands Organisation for Scientific Research (NWO).

Roles of protocol contributors

FH and JvdW are principal investigators and designed the LADI study. WK provided statistical expertise in clinical trial design. AdV, DdJ, RP, LS critically reviewed the study design. Study coordinators LS and RP ensure daily study management. LS and RP drafted the manuscript and all authors read, revised and approved the final manuscript.

Disclosures

L.J.T. Smits has nothing to disclose.

R.W.M. Pauwels has nothing to disclose.

W. Kievit has nothing to disclose.

D.J. de Jong received consulting fees from Synthon Pharma, Abbvie, and MSD, and travel fees from Falk Pharma, Takeda, Abbvie, MSD, Ferring, Vifor Pharma, and Cablon Medical.

A.C. de Vries has participated in advisory board and/or received financial compensation from the following companies: Jansen, Takeda, Abbvie and Tramedico.

F. Hoentjen has served on advisory boards or as speaker for Abbvie, Janssen-Cilag, MSD, Takeda, Celltrion, Teva, Sandoz and Dr Falk, and received unrestricted funding from Dr Falk, Janssen-Cilag, Abbvie and Celgene.

C.J. van der Woude received grant support from Falk Benelux and Pfizer; received speaker fees from AbbVie, Takeda, Ferring, Dr. Falk Pharma, Hospira, Pfizer; and served as a consultant for AbbVie, MSD, Takeda, Celgene, Mundipharma and Janssen.

For peer review only

Coordinating centres

Radboud University Medical Centre, Department of Gastroenterology and Hepatology, Nijmegen, The Netherlands

Erasmus Medical Centre, Department of Gastroenterology and Hepatology, Rotterdam, The Netherlands

Sponsor

Radboud University Medical Centre

P.O. Box 9101

6500 HB Nijmegen, The Netherlands

Principal investigators

F. Hoentjen, MD PhD & C.J. van der Woude, MD PhD

Participating centres and lead investigators

1. Albert Schweitzer Hospital, Dordrecht, The Netherlands; F.H.J. Wolfhagen, MD PhD, Department of Gastroenterology
2. Amphia Hospital, Breda, The Netherlands; A.G.L. Bodelier, MD PhD, Department of Gastroenterology
3. Amsterdam University Medical Centre-location AMC, Amsterdam, The Netherlands; M. Löwenberg, MD PhD, Department of Gastroenterology
4. Amsterdam University Medical Centre-location VUmc, Amsterdam, The Netherlands; N. de Boer, MD PhD, Department of Gastroenterology
5. Bernhoven Hospital, Uden, The Netherlands; I.A.M. Gisbertz, MD PhD, Department of Gastroenterology
6. Canisius-Wilhelmina Hospital, Nijmegen, The Netherlands; A.C.I.T.L. Tan, MD PhD, Department of Gastroenterology
7. Erasmus Medical Centre, Rotterdam, The Netherlands; C.J. van der Woude, MD PhD, Department of Gastroenterology
8. Elisabeth-Tweesteden Hospital, Tilburg, The Netherlands; M.W.M.D. Lutgens, MD PhD, Department of Gastroenterology
9. Flevoziekenhuis Hospital, Almere, The Netherlands; R.C. Mallant-Hent, MD PhD, Department of Gastroenterology
10. Franciscus Gasthuis & Vlietland, Rotterdam, The Netherlands; R.L. West, MD PhD, Department of Gastroenterology
11. IJsselland Hospital, Capelle aan den IJssel, The Netherlands; I. Schot, MD, & K.Robbers, MD, Department of Gastroenterology
12. Ikazia Hospital, Rotterdam, The Netherlands; P.C.J. ter Borg, MD PhD, Department of Gastroenterology
13. Jeroen Bosch Hospital, Den Bosch, The Netherlands; T. Römkens, MD PhD, Department of Gastroenterology
14. Leiden University Medical Centre, Leiden, The Netherlands; A.E. van der Meulen, MD PhD, Department of Gastroenterology
15. Maastricht University Medical Centre+, Maastricht, The Netherlands; M. Pierik, MD Phd, Department of Gastroenterology

16. Maxima Medical Centre, Veldhoven/Eindhoven, The Netherlands; P.J. Boekema, MD PhD, Department of Gastroenterology
17. Medisch Spectrum Twente, Enschede, The Netherlands; M.G.V.M. Russel, MD PhD, Department of Gastroenterology
18. Onze Lieve Vrouwe Gasthuis, Amsterdam, The Netherlands; J. Jansen, MD PhD, Department of Gastroenterology
19. Radboud University Medical Centre, Nijmegen, The Netherlands; F. Hoentjen, MD PhD, Department of Gastroenterology
20. Reinier de Graaf Hospital, Delft, The Netherlands; S.V. Jansen, MD, Department of Gastroenterology
21. Spaarne Gasthuis, Haarlem, The Netherlands; J.P. Kuijvenhoven, MD PhD, Department of Gastroenterology
22. University Medical Centre Utrecht, Utrecht, The Netherlands; B .Oldenburg, MD PhD, Department of Gastroenterology
23. Zuyderland Hospital, Heerlen/Sittard, The Netherlands; M.J.L. Romberg-Camps, MD PhD, Department of Gastroenterology

Data safety monitoring board

R.J.M. Brüggemann, PharmD PhD, Department of Pharmacy, Radboud University Medical Centre, Nijmegen, The Netherlands (chair)

J. in t Hout, PhD, Department for Health Evidence, Radboud University Medical Centre, Nijmegen, The Netherlands

R.J. de Knegt, MD PhD, Department of Gastroenterology and Hepatology, Erasmus Medical Centre, Rotterdam, The Netherlands

Independent expert

E.T.T.L. Tjwa, MD PhD, Department of Gastroenterology and Hepatology, Radboud University Medical Centre, Nijmegen, The Netherlands

Availability of data and materials

The dataset generated during the LADI study is available on reasonable request.

For peer review only

FIGURE LEGENDS

Figure 1. Protocolised treatment recommendation in case of disease flare

T0 = start of possible disease flare, which can occur at any time during follow-up, T2 = 2 weeks after T0, T6-8 = 6-8 weeks after T0. Lab tests include hemoglobin, leukocytes, thrombocytes, albumin, C-reactive protein, calprotectin

Figure 2. Schematic presentation of the trial design.

ADA = adalimumab, W0 = week 0, W6 = week 6, etc. Lab tests include hemoglobin, leukocytes, thrombocytes, albumin, C-reactive protein, calprotectin

For peer review only

REFERENCES

1. Torres J, Mehandru S, Colombel JF, et al. Crohn's disease. *Lancet* 2017;389(10080):1741-55. doi: 10.1016/s0140-6736(16)31711-1 [published Online First: 2016/12/05]
2. Colombel JF, Sandborn WJ, Rutgeerts P, et al. Adalimumab for maintenance of clinical response and remission in patients with Crohn's disease: the CHARM trial. *Gastroenterology* 2007;132(1):52-65. doi: 10.1053/j.gastro.2006.11.041
3. Sandborn WJ, Hanauer SB, Rutgeerts P, et al. Adalimumab for maintenance treatment of Crohn's disease: results of the CLASSIC II trial. *Gut* 2007;56(9):1232-9. doi: 10.1136/gut.2006.106781
4. Hanauer SB, Sandborn WJ, Rutgeerts P, et al. Human anti-tumor necrosis factor monoclonal antibody (adalimumab) in Crohn's disease: the CLASSIC-I trial. *Gastroenterology* 2006;130(2):323-33; quiz 591. doi: 10.1053/j.gastro.2005.11.030 [published Online First: 2006/02/14]
5. Gomollon F, Dignass A, Annese V, et al. 3rd European Evidence-based Consensus on the Diagnosis and Management of Crohn's Disease 2016: Part 1: Diagnosis and Medical Management. *Journal of Crohn's & colitis* 2017;11(1):3-25. doi: 10.1093/ecco-jcc/jjw168 [published Online First: 2016/09/24]
6. Burmester GR, Panaccione R, Gordon KB, et al. Adalimumab: Long-term safety in 23 458 patients from global clinical trials in rheumatoid arthritis, juvenile idiopathic arthritis, ankylosing spondylitis, psoriatic arthritis, psoriasis and Crohn's disease. *Ann Rheum Dis* 2013;72(4):517-24. doi: 10.1136/annrheumdis-2011-201244
7. Osterman MT, Sandborn WJ, Colombel JF, et al. Increased risk of malignancy with adalimumab combination therapy, compared with monotherapy, for Crohn's disease. *Gastroenterology* 2014;146(4):941-9. doi: 10.1053/j.gastro.2013.12.025 [published Online First: 2013/12/24]
8. Pariente B, Laharie D. Review article: why, when and how to de-escalate therapy in inflammatory bowel diseases. *Alimentary pharmacology & therapeutics* 2014;40(4):338-53. doi: 10.1111/apt.12838 [published Online First: 2014/06/25]
9. D'Haens G, Reinisch W, Panaccione R, et al. Lymphoma Risk and Overall Safety Profile of Adalimumab in Patients With Crohn's Disease With up to 6 Years of Follow-Up in the Pyramid Registry. *The American journal of gastroenterology* 2018;113(6):872-82. doi: 10.1038/s41395-018-0098-4 [published Online First: 2018/06/06]
10. Colombel JF, Sandborn WJ, Reinisch W, et al. Long-term safety of adalimumab in clinical trials in adult patients with Crohn's disease or ulcerative colitis. *Alimentary pharmacology & therapeutics* 2018;47(2):219-28. doi: 10.1111/apt.14420 [published Online First: 2017/11/22]
11. McKenna MR, Stobaugh DJ, Deepak P. Melanoma and non-melanoma skin cancer in inflammatory bowel disease patients following tumor necrosis factor-alpha inhibitor monotherapy and in combination with thiopurines: analysis of the Food and Drug Administration Adverse Event Reporting System. *Journal of gastrointestinal and liver diseases : JGLD* 2014;23(3):267-71. doi: 10.15403/jgld.2014.1121.233.mrmk [published Online First: 2014/10/01]
12. Long MD, Martin CF, Pipkin CA, et al. Risk of melanoma and nonmelanoma skin cancer among patients with inflammatory bowel disease. *Gastroenterology* 2012;143(2):390-99.e1. doi: 10.1053/j.gastro.2012.05.004 [published Online First: 2012/05/16]
13. van der Valk ME, Mangen MJ, Leenders M, et al. Healthcare costs of inflammatory bowel disease have shifted from hospitalisation and surgery towards anti-TNFalpha therapy: results from the COIN study. *Gut* 2014;63(1):72-9. doi: 10.1136/gutjnl-2012-303376 [published Online First: 2012/11/09]
14. van der Valk ME, Mangen MJ, Severs M, et al. Evolution of Costs of Inflammatory Bowel Disease over Two Years of Follow-Up. *PLoS One* 2016;11(4):e0142481. doi: 10.1371/journal.pone.0142481 [published Online First: 2016/04/23]
15. van den Heuvel TRA, Jeuring SFG, Zeegers MP, et al. A 20-Year Temporal Change Analysis in Incidence, Presenting Phenotype and Mortality, in the Dutch IBDSL Cohort-Can Diagnostic Factors Explain the Increase in IBD Incidence? *Journal of*

For peer review only - http://bmjopen.bmj.com/site/about/guidelines.xhtml

Crohn's & colitis 2017;11(10):1169-79. doi: 10.1093/ecco-jcc/jjx055 [published Online First: 2017/04/22]
16. Colombel JF, Panaccione R, Bossuyt P, et al. Effect of tight control management on Crohn's disease (CALM): a multicentre, randomised, controlled phase 3 trial. *Lancet* 2018;390(10114):2779-89. doi: 10.1016/s0140-6736(17)32641-7 [published Online First: 2017/11/04]
17. Brodszky V, Rencz F, Pentek M, et al. A budget impact model for biosimilar infliximab in Crohn's disease in Bulgaria, the Czech Republic, Hungary, Poland, Romania, and Slovakia. *Expert review of pharmacoeconomics & outcomes research* 2016;16(1):119-25. doi: 10.1586/14737167.2015.1067142 [published Online First: 2015/07/15]
18. Kuenzig ME, Benchimol EI, Lee L, et al. The Impact of Inflammatory Bowel Disease in Canada 2018: Direct Costs and Health Services Utilization. *Journal of the Canadian Association of Gastroenterology* 2019;2(Suppl 1):S17-s33. doi: 10.1093/jcag/gwy055 [published Online First: 2019/07/12]
19. Bots S, Hoekman DR, Benninga MA, et al. Patterns of anti-TNF use and associated treatment outcomes in inflammatory bowel disease patients: Results from an analysis of dutch health insurance claims data. *Gastroenterology* 2017;152(5):S58.
20. Louis E, Mary JY, Vernier-Massouille G, et al. Maintenance of remission among patients with Crohn's disease on antimetabolite therapy after infliximab therapy is stopped. *Gastroenterology* 2012;142(1):63-70 e5; quiz e31. doi: 10.1053/j.gastro.2011.09.034
21. van Herwaarden N, van der Maas A, Minten MJ, et al. Disease activity guided dose reduction and withdrawal of adalimumab or etanercept compared with usual care in rheumatoid arthritis: open label, randomised controlled, non-inferiority trial. *Bmj* 2015;350:h1389. doi: 10.1136/bmj.h1389
22. Kievit W, van Herwaarden N, van den Hoogen FH, et al. Disease activity-guided dose optimisation of adalimumab and etanercept is a cost-effective strategy compared with non-tapering tight control rheumatoid arthritis care: analyses of the DRESS study. *Annals of the rheumatic diseases* 2016;75(11):1939-44. doi: 10.1136/annrheumdis-2015-208317 [published Online First: 2016/01/15]
23. Papamichael K, Van Stappen T, Jairath V, et al. Review article: pharmacological aspects of anti-TNF biosimilars in inflammatory bowel diseases. *Alimentary pharmacology & therapeutics* 2015;42(10):1158-69. doi: 10.1111/apt.13402 [published Online First: 2015/09/15]
24. Tilg H, Moschen A, Kaser A. Mode of function of biological anti-TNF agents in the treatment of inflammatory bowel diseases. *Expert Opin Biol Ther* 2007;7(7):1051-9. doi: 10.1517/14712598.7.7.1051 [published Online First: 2007/08/02]
25. Pouillon L, Lamoureux A, Pineton de Chambrun G, et al. Dose de-escalation to adalimumab 40mg every three weeks in patients with inflammatory bowel disease-A multicenter, retrospective, observational study. *Digestive and liver disease : official journal of the Italian Society of Gastroenterology and the Italian Association for the Study of the Liver* 2019;51(2):236-41. doi: 10.1016/j.dld.2018.10.022 [published Online First: 2018/12/07]
26. Van Steenbergen S, Bian S, Vermeire S, et al. Dose de-escalation to adalimumab 40 mg every 3 weeks in patients with Crohn's disease - a nested case-control study. *Alimentary pharmacology & therapeutics* 2017;45(7):923-32. doi: 10.1111/apt.13964 [published Online First: 2017/02/07]
27. Chan AW, Tetzlaff JM, Gotzsche PC, et al. SPIRIT 2013 explanation and elaboration: guidance for protocols of clinical trials. *Bmj* 2013;346:e7586. doi: 10.1136/bmj.e7586 [published Online First: 2013/01/11]
28. ECCO obo, Gionchetti P, Gomollón F, et al. 3rd European Evidence-based Consensus on the Diagnosis and Management of Crohn's Disease 2016: Part 1: Diagnosis and Medical Management. *Journal of Crohn's and Colitis* 2016;11(1):3-25. doi: 10.1093/ecco-jcc/jjw168
29. van Bodegraven AA, van Everdingen JJ, Dijkstra G, et al. [Guideline 'Diagnosis and treatment of inflammatory bowel disease in adults'. I. Diagnosis and treatment]. *Nederlands tijdschrift voor geneeskunde* 2010;154:A1899. [published Online First: 2010/10/30]

30. Irvine EJ, Zhou Q, Thompson AK. The Short Inflammatory Bowel Disease Questionnaire: a quality of life instrument for community physicians managing inflammatory bowel disease. CCRPT Investigators. Canadian Crohn's Relapse Prevention Trial. *The American journal of gastroenterology* 1996;91(8):1571-8. [published Online First: 1996/08/01]
31. Khanna R, Zou G, D'Haens G, et al. A retrospective analysis: the development of patient reported outcome measures for the assessment of Crohn's disease activity. *Alimentary pharmacology & therapeutics* 2015;41(1):77-86. doi: 10.1111/apt.13001 [published Online First: 2014/10/29]
32. Janssen MF, Pickard AS, Golicki D, et al. Measurement properties of the EQ-5D-5L compared to the EQ-5D-3L across eight patient groups: a multi-country study. *Quality of life research : an international journal of quality of life aspects of treatment, care and rehabilitation* 2013;22(7):1717-27. doi: 10.1007/s11136-012-0322-4
33. EuroQol G. EuroQol--a new facility for the measurement of health-related quality of life. *Health policy* 1990;16(3):199-208.
34. Flight L, Julious SA. Practical guide to sample size calculations: non-inferiority and equivalence trials. *Pharmaceutical statistics* 2016;15(1):80-9. doi: 10.1002/pst.1716 [published Online First: 2015/11/26]
35. Karmiris K, Paintaud G, Noman M, et al. Influence of trough serum levels and immunogenicity on long-term outcome of adalimumab therapy in Crohn's disease. *Gastroenterology* 2009;137(5):1628-40. doi: 10.1053/j.gastro.2009.07.062 [published Online First: 2009/08/12]
36. Jorgensen KK, Olsen IC, Goll GL, et al. Switching from originator infliximab to biosimilar CT-P13 compared with maintained treatment with originator infliximab (NOR-SWITCH): a 52-week, randomised, double-blind, non-inferiority trial. *Lancet* 2017;389(10086):2304-16. doi: 10.1016/s0140-6736(17)30068-5 [published Online First: 2017/05/16]
37. Burmester GR, Panaccione R, Gordon KB, et al. Adalimumab: long-term safety in 23 458 patients from global clinical trials in rheumatoid arthritis, juvenile idiopathic arthritis, ankylosing spondylitis, psoriatic arthritis, psoriasis and Crohn's disease. *Annals of the rheumatic diseases* 2013;72(4):517-24. doi: 10.1136/annrheumdis-2011-201244 [published Online First: 2012/05/09]
38. Kahan BC, Morris TP. Reporting and analysis of trials using stratified randomisation in leading medical journals: review and reanalysis. *Bmj* 2012;345:e5840. doi: 10.1136/bmj.e5840 [published Online First: 2012/09/18]
39. E9 I. Statistical principles for clinical trials. Note for guidance on statistical principles for clinical trials. *EMA*;CPMP/ICH/363/96
40. Gisbert JP, Marín AC, Chaparro M. The risk of relapse after Anti-TNF discontinuation in inflammatory bowel disease: Systematic review and meta-analysis. *Am J Gastroenterol* 2016;111(5):632-47. doi: 10.1038/ajg.2016.54
41. Kennedy NA, Warner B, Johnston E, et al. Anti-TNF withdrawal in IBD: Relapse and recapture rates and predictive factors from 160 patients in a pan-UK study. *J Crohn's Colitis* 2015;9:S41-S42. doi: 10.1093/ecco-jcc/jju027
42. Louis E, Mary JY, Verniermassouille G, et al. Maintenance of remission among patients with Crohn's disease on antimetabolite therapy after infliximab therapy is stopped. *Gastroenterology* 2012;142(1):63-70.e5. doi: 10.1053/j.gastro.2011.09.034
43. Casanova MJ, Chaparro M, García-Sánchez V, et al. Evolution after Anti-TNF Discontinuation in Patients with Inflammatory Bowel Disease: A Multicenter Long-Term Follow-Up Study. *Am J Gastroenterol* 2017;112(1):120-31. doi: 10.1038/ajg.2016.569
44. Ma C, Battat R, Parker CE, et al. Update on C-reactive protein and fecal calprotectin: are they accurate measures of disease activity in Crohn's disease? *Expert Rev Gastroenterol Hepatol* 2019:1-12. doi: 10.1080/17474124.2019.1563481 [published Online First: 2019/02/23]
45. D'Haens G, Ferrante M, Vermeire S, et al. Fecal calprotectin is a surrogate marker for endoscopic lesions in inflammatory bowel disease. *Inflammatory bowel diseases* 2012;18(12):2218-24. doi: 10.1002/ibd.22917

46. Kennedy NA, Jones GR, Plevris N, et al. Association Between Level of Fecal Calprotectin and Progression of Crohn's Disease. *Clin Gastroenterol Hepatol* 2019 doi: 10.1016/j.cgh.2019.02.017 [published Online First: 2019/02/18]
47. Ferreiro-Iglesias R, Barreiro-de Acosta M, Lorenzo-Gonzalez A, et al. Accuracy of Consecutive Fecal Calprotectin Measurements to Predict Relapse in Inflammatory Bowel Disease Patients Under Maintenance With Anti-TNF Therapy: A Prospective Longitudinal Cohort Study. *J Clin Gastroenterol* 2018;52(3):229-34.
48. Atalay S, van den Reek J, van Vugt LJ, et al. Tight controlled dose reduction of biologics in psoriasis patients with low disease activity: a randomized pragmatic non-inferiority trial. *BMC dermatology* 2017;17(1):6. doi: 10.1186/s12895-017-0057-6 [published Online First: 2017/05/10]

For peer review only

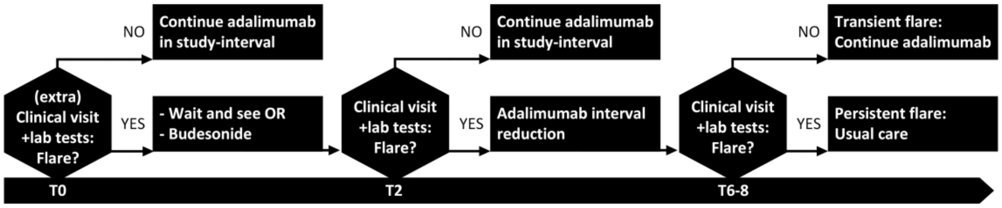

Figure 1. Protocolised treatment recommendation in case of disease flare
T0 = start of possible disease flare, which can occur at any time during follow-up, T2 = 2 weeks after T0, T6-8 = 6-8 weeks after T0. Lab tests include hemoglobin, leukocytes, thrombocytes, albumin, C-reactive protein, calprotectin

283x57mm (300 x 300 DPI)

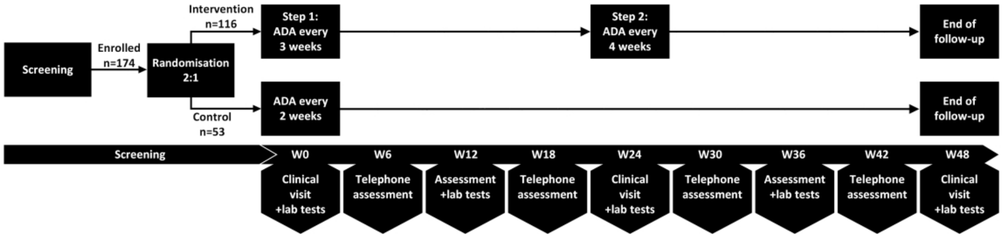

Figure 2. Schematic presentation of the trial design.
ADA = adalimumab, W0 = week 0, W6 = week 6, etc. Lab tests include hemoglobin, leukocytes, thrombocytes, albumin, C-reactive protein, calprotectin

281x66mm (300 x 300 DPI)

Supplementary file 1: SPIRIT checklist

Section/item	Item No	Description	Addressed on page number
**Administrative information**			
Title	1	Descriptive title identifying the study design, population, interventions, and, if applicable, trial acronym	1
Trial registration	2a	Trial identifier and registry name. If not yet registered, name of intended registry	8, abstract
	2b	All items from the World Health Organization Trial Registration Data Set	Throughout entire protocol
Protocol version	3	Date and version identifier	8
Funding	4	Sources and types of financial, material, and other support	19
Roles and responsibilities	5a	Names, affiliations, and roles of protocol contributors	20
	5b	Name and contact information for the trial sponsor	20
	5c	Role of study sponsor and funders, if any, in study design; collection, management, analysis, and interpretation of data; writing of the report; and the decision to submit the report for publication, including whether they will have ultimate authority over any of these activities	20
	5d	Composition, roles, and responsibilities of the coordinating centre, steering committee, endpoint adjudication committee, data management team, and other individuals or groups overseeing the trial, if applicable (see Item 21a for data monitoring committee)	20-22
**Introduction**			
Background and rationale	6a	Description of research question and justification for undertaking the trial, including summary of relevant studies (published and unpublished) examining benefits and harms for each intervention	4-5
	6b	Explanation for choice of comparators	4-5
Objectives	7	Specific objectives or hypotheses	5-6

For peer review only

Trial design	8	Description of trial design including type of trial (eg, parallel group, crossover, factorial, single group), allocation ratio, and framework (eg, superiority, equivalence, noninferiority, exploratory)	8
**Methods: Participants, interventions, and outcomes**			
Study setting	9	Description of study settings (eg, community clinic, academic hospital) and list of countries where data will be collected. Reference to where list of study sites can be obtained	8, 21-22
Eligibility criteria	10	Inclusion and exclusion criteria for participants. If applicable, eligibility criteria for study centres and individuals who will perform the interventions (eg, surgeons, psychotherapists)	9
Interventions	11a	Interventions for each group with sufficient detail to allow replication, including how and when they will be administered	8-10, Figure 1, Table 1
	11b	Criteria for discontinuing or modifying allocated interventions for a given trial participant (eg, drug dose change in response to harms, participant request, or improving/worsening disease)	10, Figure 2
	11c	Strategies to improve adherence to intervention protocols, and any procedures for monitoring adherence (eg, drug tablet return, laboratory tests)	11
	11d	Relevant concomitant care and interventions that are permitted or prohibited during the trial	9-10
Outcomes	12	Primary, secondary, and other outcomes, including the specific measurement variable (eg, systolic blood pressure), analysis metric (eg, change from baseline, final value, time to event), method of aggregation (eg, median, proportion), and time point for each outcome. Explanation of the clinical relevance of chosen efficacy and harm outcomes is strongly recommended	6-7, 9-12
Participant timeline	13	Time schedule of enrolment, interventions (including any run-ins and washouts), assessments, and visits for participants. A schematic diagram is highly recommended (see Figure)	Figure 1
Sample size	14	Estimated number of participants needed to achieve study objectives and how it was determined, including clinical and statistical assumptions supporting any sample size calculations	13
Recruitment	15	Strategies for achieving adequate participant enrolment to reach target sample size	8
**Methods: Assignment of interventions (for controlled trials)**			

Allocation:			
Sequence generation	16a	Method of generating the allocation sequence (eg, computer-generated random numbers), and list of any factors for stratification. To reduce predictability of a random sequence, details of any planned restriction (eg, blocking) should be provided in a separate document that is unavailable to those who enrol participants or assign interventions	13
Allocation concealment mechanism	16b	Mechanism of implementing the allocation sequence (eg, central telephone; sequentially numbered, opaque, sealed envelopes), describing any steps to conceal the sequence until interventions are assigned	13
Implementation	16c	Who will generate the allocation sequence, who will enrol participants, and who will assign participants to interventions	13
Blinding (masking)	17a	Who will be blinded after assignment to interventions (eg, trial participants, care providers, outcome assessors, data analysts), and how	13
	17b	If blinded, circumstances under which unblinding is permissible, and procedure for revealing a participant's allocated intervention during the trial	Not applicable: unblinded
**Methods: Data collection, management, and analysis**			
Data collection methods	18a	Plans for assessment and collection of outcome, baseline, and other trial data, including any related processes to promote data quality (eg, duplicate measurements, training of assessors) and a description of study instruments (eg, questionnaires, laboratory tests) along with their reliability and validity, if known. Reference to where data collection forms can be found, if not in the protocol	9-11
	18b	Plans to promote participant retention and complete follow-up, including list of any outcome data to be collected for participants who discontinue or deviate from intervention protocols	-
Data management	19	Plans for data entry, coding, security, and storage, including any related processes to promote data quality (eg, double data entry; range checks for data values). Reference to where details of data management procedures can be found, if not in the protocol	8
Statistical methods	20a	Statistical methods for analysing primary and secondary outcomes. Reference to where other details of the statistical analysis plan can be found, if not in the protocol	14-15

	20b	Methods for any additional analyses (eg, subgroup and adjusted analyses)	14-15
	20c	Definition of analysis population relating to protocol non-adherence (eg, as randomised analysis), and any statistical methods to handle missing data (eg, multiple imputation)	-
**Methods: Monitoring**			
Data monitoring	21a	Composition of data monitoring committee (DMC); summary of its role and reporting structure; statement of whether it is independent from the sponsor and competing interests; and reference to where further details about its charter can be found, if not in the protocol. Alternatively, an explanation of why a DMC is not needed	8, 22
	21b	Description of any interim analyses and stopping guidelines, including who will have access to these interim results and make the final decision to terminate the trial	Not applicable
Harms	22	Plans for collecting, assessing, reporting, and managing solicited and spontaneously reported adverse events and other unintended effects of trial interventions or trial conduct	10
Auditing	23	Frequency and procedures for auditing trial conduct, if any, and whether the process will be independent from investigators and the sponsor	Not applicable
**Ethics and dissemination**			
Research ethics approval	24	Plans for seeking research ethics committee/institutional review board (REC/IRB) approval	8
Protocol amendments	25	Plans for communicating important protocol modifications (eg, changes to eligibility criteria, outcomes, analyses) to relevant parties (eg, investigators, REC/IRBs, trial participants, trial registries, journals, regulators)	8 standard according to Dutch Medical Research Involving Human Subjects Act (WMO)
Consent or assent	26a	Who will obtain informed consent or assent from potential trial participants or authorised surrogates, and how (see Item 32)	8 standard according to Dutch Medical Research Involving Human Subjects Act (WMO)
	26b	Additional consent provisions for collection and use of participant data and biological specimens in ancillary studies, if applicable	Not applicable
Confidentiality	27	How personal information about potential and enrolled participants will be collected, shared, and maintained in order to protect confidentiality before, during, and after the trial	8

ew only

Declaration of interests	28	Financial and other competing interests for principal investigators for the overall trial and each study site	20
Access to data	29	Statement of who will have access to the final trial dataset, and disclosure of contractual agreements that limit such access for investigators	-
Ancillary and post-trial care	30	Provisions, if any, for ancillary and post-trial care, and for compensation to those who suffer harm from trial participation	-
Dissemination policy	31a	Plans for investigators and sponsor to communicate trial results to participants, healthcare professionals, the public, and other relevant groups (eg, via publication, reporting in results databases, or other data sharing arrangements), including any publication restrictions	No publication restrictions
	31b	Authorship eligibility guidelines and any intended use of professional writers	Not applicalble
	31c	Plans, if any, for granting public access to the full protocol, participant-level dataset, and statistical code	-
**Appendices**			
Informed consent materials	32	Model consent form and other related documentation given to participants and authorised surrogates	Supplementary file B
Biological specimens	33	Plans for collection, laboratory evaluation, and storage of biological specimens for genetic or molecular analysis in the current trial and for future use in ancillary studies, if applicable	Not applicable

For peer review only

Supplementary file 2: Subject Consent Form

Step-by-step extension of the adalimumab interval in patients with Crohn's disease

- I have read the subject information form. I was also able to ask questions. My questions have been answered to my satisfaction. I had enough time to decide whether to participate.
- I know that participation is voluntary. I know that I may decide at any time not to participate after all or to withdraw from the study. I do not need to give a reason for this.
- I give permission for my GP to be informed that I am participating in this study
- I know that some people may have access to all my data to verify the study. These people are listed in this information sheet. I consent to the inspection by them.
- I give permission for sending my HealthBeacon data to the study team.
- I agree that my GP and treating specialist will be informed of coincidental findings that (may) be of interest for my health.
- I give permission for the collection and use of my data and body material to answer the research question in this study.
- I give permission for keeping my data at the research location for 25 years.
- I give permission for registration of observational data during 2 years after the study period.
- I **do / do not*** consent to keeping my bodily material 15 years after this study and to use this later for other research, as indicated in the information sheet.
- I **do / do not*** consent to being contacted again after this study for a follow-up study.
- I want to participate in this study.

Name of study subject:

Signature: Date: __ / __ / __

I hereby declare that I have fully informed this study subject about this study.

If information comes to light during the course of the study that could affect the study subject's consent, I will inform him/her of this in a timely fashion.

Name of investigator (or his/her representative):

Signature: Date:__ / __ / __

Additional information was given by:

Name:

Job title:

Signature: Date:__ / __ / __

----

* Delete as appropriate.

For peer review only - http://bmjopen.bmj.com/site/about/guidelines.xhtml

The study subject will receive the full information sheet, together with a signed copy of the consent form.

For peer review only

For peer review only - http://bmjopen.bmj.com/site/about/guidelines.xhtml
